# Macromolecular crowding in animal component-free, xeno-free and foetal bovine serum media for human bone marrow mesenchymal stromal cell expansion and differentiation

**DOI:** 10.3389/fbioe.2023.1136827

**Published:** 2023-03-06

**Authors:** Stefanie H. Korntner, Alessia Di Nubila, Diana Gaspar, Dimitrios I. Zeugolis

**Affiliations:** ^1^ Regenerative, Modular and Developmental Engineering Laboratory (REMODEL) and Science Foundation Ireland (SFI) Centre for Research in Medical Devices (CÚRAM), University of Galway, Galway, Ireland; ^2^ Regenerative, Modular and Developmental Engineering Laboratory (REMODEL), Charles Institute of Dermatology, Conway Institute of Biomolecular and Biomedical Research and School of Mechanical and Materials Engineering, University College Dublin, Dublin, Ireland

**Keywords:** macromolecular crowding, foetal bovine serum, animal component-free media, xeno-free media, mesenchymal stromal cell expansion, mesenchymal stromal cell differentiation

## Abstract

**Background:** Cell culture media containing undefined animal-derived components and prolonged *in vitro* culture periods in the absence of native extracellular matrix result in phenotypic drift of human bone marrow stromal cells (hBMSCs).

**Methods:** Herein, we assessed whether animal component-free (ACF) or xeno-free (XF) media formulations maintain hBMSC phenotypic characteristics more effectively than foetal bovine serum (FBS)-based media. In addition, we assessed whether tissue-specific extracellular matrix, induced *via* macromolecular crowding (MMC) during expansion and/or differentiation, can more tightly control hBMSC fate.

**Results:** Cells expanded in animal component-free media showed overall the highest phenotype maintenance, as judged by cluster of differentiation expression analysis. Contrary to FBS media, ACF and XF media increased cellularity over time in culture, as measured by total DNA concentration. While MMC with Ficoll™ increased collagen deposition of cells in FBS media, FBS media induced significantly lower collagen synthesis and/or deposition than the ACF and XF media. Cells expanded in FBS media showed higher adipogenic differentiation than ACF and XF media, which was augmented by MMC with Ficoll™ during expansion. Similarly, Ficoll™ crowding also increased chondrogenic differentiation. Of note, donor-to-donor variability was observed for collagen type I deposition and trilineage differentiation capacity of hBMSCs.

**Conclusion:** Collectively, our data indicate that appropriate screening of donors, media and supplements, in this case MMC agent, should be conducted for the development of clinically relevant hBMSC medicines.

## 1 Introduction

Mesenchymal stromal cells (MSCs) hold great potential for therapeutic and reparative use in tissue engineering and regenerative medicine due to their self-renewal, multipotency and immunomodulatory properties (Deans and Moseley, 2000; Di Nicola et al., 2002; Jorgensen et al., 2003). Regarding clinical translation of MSC medicines, animal-derived cell culture media components (i.e., animal sera) raise safety concerns related to xenogeneic contaminations and disease transfer through pathogens (e.g., *mycoplasma*, viruses and prions) (Selvaggi et al., 1997; Tekkatte et al., 2011; Hawkes, 2015; van der Valk, 2022). Also, antibodies against bovine antigens (when foetal bovine serum, FBS, is used, which is the most widely used serum in cell culture) may be elicited by repeated administration of cells, which will in turn directly affect the safety and efficacy of cell-based treatments to patients (Horwitz et al., 2002; Sundin et al., 2007). Moreover, the undefined composition of FBS results in inconsistent batch-to-batch performance, low reproducibility of experiments and ultimately jeopardises the therapeutic potential of MSC therapies (Honn et al., 1975; Heiskanen et al., 2007). As a tight control of cell behaviour *in vitro* is imperative for intended clinical use, recent efforts have been directed towards the development of more defined xeno-free (XF) and/or animal component-free (ACF) media formulations for translational research, development and regulatory compliant MSC medicines (Chase et al., 2012; Jung et al., 2012; Kinzebach and Bieback, 2013). Per definition, both XF and ACF media cannot contain animal-derived proteins or serum. While ACF media is entirely free of animal- and human-derived components and all elements are therefore chemically defined, XF media can contain human-derived supplements (de Soure et al., 2016; Karnieli et al., 2017).

Another limiting factor in the clinical translation of MSC therapies, especially in the case of autologous therapies, is the prolonged *in vitro* expansion required to reach the high cell numbers needed for therapeutic effects, which is associated with phenotype, immunomodulatory capability and therapeutic losses (Bara et al., 2014; Elgaz et al., 2019; L'Heureux et al., 2006; Siddappa et al., 2007; Whitfield et al., 2013; Yao et al., 2006; Zhang et al., 2015). In artificial *in vitro* cell culture systems, cells are grown in liquid media in planar 2D cultures, which poorly resemble the native *in vivo* scenario where cells reside in a dense 3D microenvironment, in direct contact with the extracellular matrix (ECM). *In vivo*, the dynamic reciprocity between cells and their surrounding ECM determines their fate and function (Roskelley and Bissell, 1995; Thorne et al., 2015; van Helvert et al., 2018). Similarly, the presence of tissue-specific ECM has been shown to facilitate cell phenotype maintenance *in vitro* (Pei et al., 2011; Cheng et al., 2014; Gattazzo et al., 2014; Yang et al., 2018a). In eukaryotic cell culture systems, macromolecular crowding (MMC), following the principles of excluded volume effect, enhances and accelerates tissue-specific ECM deposition (Raghunath and Zeugolis, 2021; Tsiapalis and Zeugolis, 2021; Zeugolis, 2021), a phenomenon that has been well documented in both differentiated (Lareu et al., 2007; Satyam et al., 2014; Kumar et al., 2015a; Kumar et al., 2015b; Satyam et al., 2016; Kumar et al., 2018; Gaspar et al., 2019; Shendi et al., 2019; Tsiapalis et al., 2021) and progenitor cell cultures (Zeiger et al., 2012; Prewitz et al., 2015; Cigognini et al., 2016; Lee et al., 2016; Patrikoski et al., 2017; Graceffa and Zeugolis, 2019; De Pieri et al., 2020). However, to-date, only one study has assessed the influence of MMC in xeno-free and/or serum-free media formulations using human adipose-derived mesenchymal stromal cells (Patrikoski et al., 2017).

**FIGURE 1:**
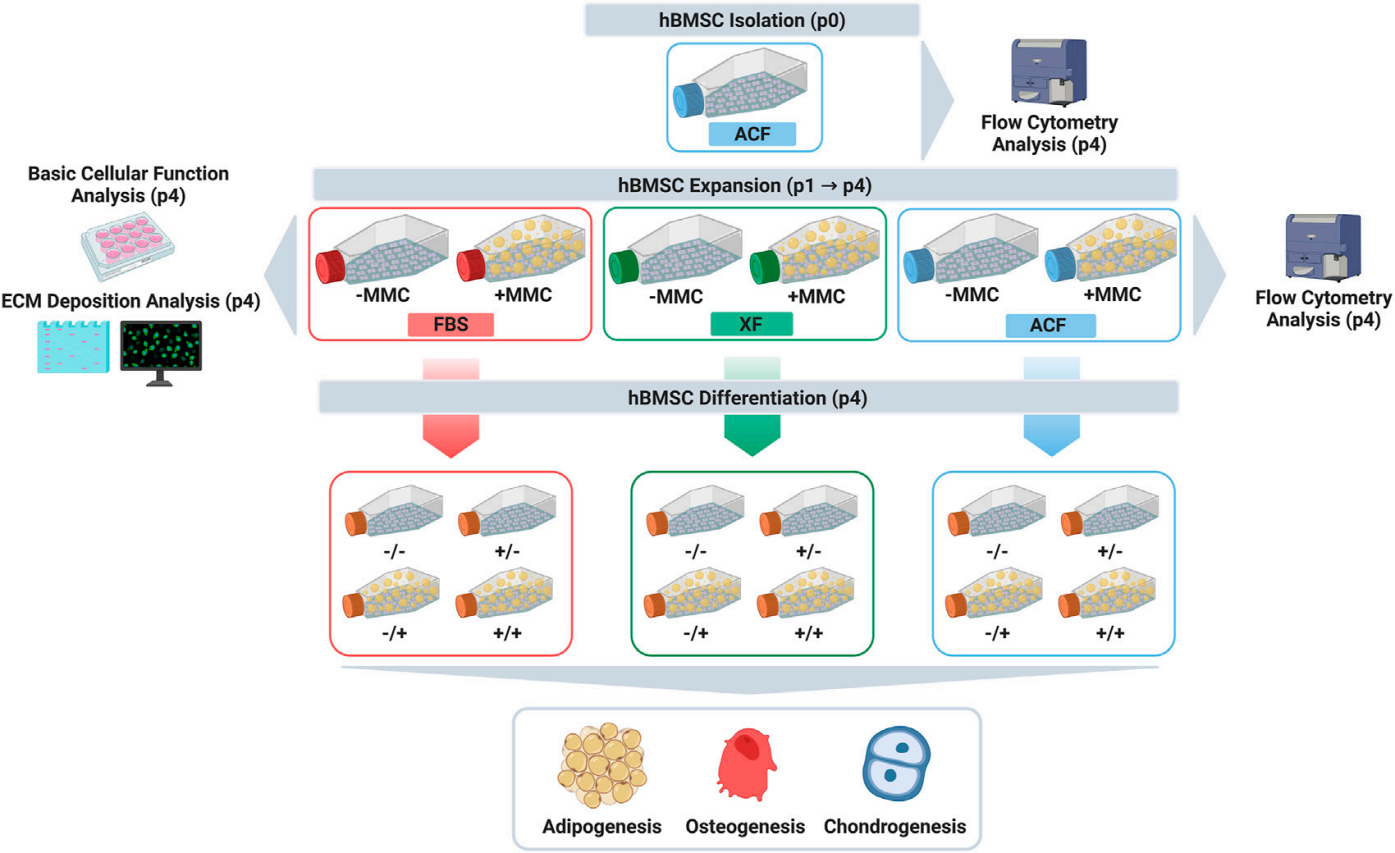
Study design. Created with BioRender.com.

Considering the above, herein we ventured to investigate the influence of MMC in FBS, XF and ACF media on human bone marrow mesenchymal stromal cell (hBMSC) expansion and differentiation. Cells from two donors were isolated in ACF media and expanded from passage 0 (p0) to passage 4 (p4) in FBS, XF and ACF media, in the absence and presence of MMC. At p4, phenotype, viability, metabolic activity, proliferation, collagen deposition and trilineage differentiation analyses were assessed (Figure 1 graphically illustrates the study design).

## 2 Materials and methods

### 2.1 Materials

Ficoll™ (Fc) 70 kDa and 400 kDa were purchased from Sigma Aldrich (Ireland). Polysucrose 1,000 kDa (Fc 1,000 kDa) was purchased from TdB Consultancy AB (Sweden). MesenCult™ ACF Plus Media and supplements were purchased from STEMCELL Technologies (United Kingdom). MSC NutriStem® XF Medium (Biological Industries) was purchased from Geneflow Ltd. (United Kingdom). Tissue culture consumables were purchased from Sarstedt (Ireland) and NUNC (Denmark). All other chemicals, cell culture media and reagents were purchased from Sigma Aldrich (Ireland), unless otherwise stated.

### 2.2 Isolation and expansion of hBMSCs

Fresh human whole bone marrow from the iliac crest of two different donors (donor 1: female, 22 years old; donor 2: male, 25 years old) was purchased from AllCells® (United States) and hBMSCs were isolated using the MesenCult™-ACF Plus medium, according to the manufacturer’s protocol. In order to provide a complete ACF culture system, the MesenCult™-ACF Plus medium was used in conjunction with ACF Cell Attachment Substrate (STEMCELL Technologies, United Kingdom) and ACF Cell Dissociation Kit (STEMCELL Technologies, United Kingdom). hBMSCs were isolated using density gradient medium separation (Lymphoprep™, STEMCELL Technologies, United Kingdom). Briefly, phosphate buffered saline (PBS) containing 2 mM ethylenediaminetetraacetic acid (EDTA) and Lymphoprep™ were added to the bone marrow sample. After centrifugation at 300 *g* for 30 min, the mononuclear cell layer was collected at the plasma/Lymphoprep™ interface and washed with cold PBS containing 2 mM EDTA. After another centrifugation step at 300 x g for 10 min, the supernatant was discarded, and the cell pellet resuspended in complete MesenCult™-ACF Plus medium. Nucleated cells were counted using 3% acetic acid with methylene blue (STEMCELL Technologies, United Kingdom) and seeded into pre-coated (ACF cell attachment substrate) culture flasks with complete MesenCult™-ACF Plus medium at a density of 50,000 freshly isolated cells/cm^2^. Flasks were incubated at 37°C until cells reached a confluency of approximately 80%. A half-medium change was performed on day 7. Cells up to this stage were considered to be at p0.

From p1 onwards, hBMSCs were subjected to MMC treatment. For MMC conditions, a Fc cocktail, composed of 10 mg/mL Fc 70 kDa, 25 mg/mL Fc 400 kDa and 2.25 mg/mL Fc 1,000 kDa [Fc cocktail was previously optimised for maximum excluded volume effect (Gaspar et al., 2019)] was used, dissolved in the respective media. Cells were expanded in MesenCult™-ACF Plus Medium, STEMCELL Technologies, United Kingdom (referred to from now on as ACF) or MSC NutriStem® XF Medium, Biological Industries, United Kingdom (referred to from now on as XF) without and with MMC, according to the manufacturer’s protocols. 1% penicillin/streptomycin (P/S) was added to both ACF and XF media formulations. For a serum-containing control, cells were expanded in alpha-Minimum Essential Medium (α-MEM) with GlutaMAX (Gibco Life Technologies, Ireland) supplemented with 10% FBS, Life Technologies, Ireland (referred to from now on as FBS), 1% (P/S), 1 ng/mL of basic fibroblast growth factor/fibroblast growth factor 2 and without and with MMC. FBS, XF and ACF media were supplemented with 100 μM L-ascorbic acid 2-phosphate sesquimagnesium salt hydrate, to induce collagen synthesis. Cells in each media (Supplementary Table S1) were expanded at 37°C in a humidified atmosphere of 5% CO_2_ until p4. hBMSCs expanded with the ACF medium, XF medium and FBS medium were detached using the ACF Cell Dissociation Kit (STEMCELL Technologies, United Kingdom), the TrypLE Select (Life Technologies) and trypsin-EDTA (Life Technologies), respectively.

### 2.3 Flow cytometry analysis

Flow cytometry (BD FACSCanto™ II, BD Biosciences, Belgium and BD Stemflow™, United Kingdom) was used to determine the immunophenotype of hBMSCs at p0 and to assess the effect of different expansion media and MMC on immunophenotype of BMSCs at p4 after 10 days of culture. The monoclonal antibodies against cluster of differentiation (CD) CD105, CD73, CD90, CD44, CD45, CD31 and CD146 and their respective isotype controls are provided in Supplementary Table S2. Briefly, cells were detached using respective detachment solutions, centrifuged and resuspended in 2% FBS in PBS. After straining using a 40 μm cell strainer, cells were counted and diluted to a concentration of 1,000,000 cells/mL in 2% FBS in PBS. Subsequently, ∼100,000 cells were placed in each tube and stained with the appropriate volume of fluorochrome-labelled antibodies for 30 min at 4°C. Cells were washed with PBS and resuspended in 2% FBS in PBS. Analysis was performed on 100,000 cells per sample and unstained cell samples were used to correct for background autofluorescence. SYTOX™ Blue Dead Cell Stain (Invitrogen, United Kingdom) was used to label and exclude dead cells. Single stained samples were used to determine the level of spectral overlap between different fluorophores and for compensation. Fluorescence minus one (FMO) controls were used to determine gating boundaries. Isotype control antibodies were used to assess the level of background staining and non-specific binding. Cells were analysed using a BD FACSCanto™ II cytometer (BD Biosciences, United Kingdom) and Median Fluorescence Intensity of hBMSCs was calculated using FlowJo® software v10 (TreeStar Inc., United States). The gating strategy was as follows: a primary gate was placed on the area vs height signal of the forward scatter (FSC-A/FSC-H) dot plot to discriminate for doublets and cell aggregates. The single cell population was identified by defining the gated population on a side scatter area signal vs a forward scatter area (SSC-A/FSC-A) signal dot plot. Single parameter histograms were generated, overlayed with respective isotype controls, and range gates were used to determine the percentage of cells expressing the individual surface markers.

### 2.4 Phase contrast microscopy analysis

To assess morphological changes of hBMSCs during cell expansion and during trilineage differentiation, cells were observed using an inverted brightfield microscope (Leica Microsystem, Germany). Phase contrast images were captured at different passages and during trilineage differentiation and were processed using ImageJ software (NIH, United States).

### 2.5 Cell viability analysis

At p4 cells were seeded at a density 25,000 cells/cm^2^, and a Live/Dead assay, with calcein AM (ThermoFisher Scientific, United Kingdom) and ethidium homodimer I (ThermoFisher Scientific, United Kingdom) stainings, was performed at day 4 and day 10 of culture, as per manufacturer’s protocol. In live cells, non-fluorescent calcein-AM is converted to green fluorescent calcein after acetoxymethyl ester hydrolysis by intracellular esterases. Ethidium homodimer I can penetrate the disrupted cell membranes of dead or dying cells and binds to DNA, producing a red fluorescence. Briefly, at each time point, cells were washed with Hank’s Balanced Salt Solution (HBSS) and a solution of calcein AM (4 μM) and ethidium homodimer I (2 μM) was added. After 30 min incubation at 37°C in a humidified atmosphere of 5% CO_2_, fluorescence images were obtained with an Olympus IX-81 inverted fluorescence microscope (Olympus Corporation, Japan). For each condition dimethyl sulfoxide treated cells were used as negative control.

### 2.6 DNA concentration analysis

At p4, cells were seeded at a density of 25,000 cells/cm^2^, and a Quant-iT™ PicoGreen® dsDNA (ThermoFisher Scientific, United Kingdom) assay was performed to quantify the amount of dsDNA present in the respective samples at day 4 and day 10 of culture, as per manufacturer’s protocol. Briefly, 250 μl of nucleic acid-free water was added to each well (24-well plate), the well plate was frozen at −80°C and three freeze-thaw cycles were performed to lyse the cells and extract the DNA. 100 μl of each DNA sample were transferred into a 96-well plate. A standard curve was generated with 0, 200, 375, 500, 1,000 and 2,000 ng/mL DNA concentrations. 100 μl of PicoGreen® reagent at 1:200 dilution in 1X Tris-EDTA buffer was added to all standards and samples. Fluorescence values (excitation: 480 nm, emission: 520 nm) were obtained with a Varioskan Flash Spectral scanning multimode reader (ThermoFisher Scientific, United Kingdom). The DNA concentration was defined as a function of the standard curve and compared at day 4 and day 10.

### 2.7 Cell metabolic activity analysis

At p4, cells were seeded at a density of 25,000 cells/cm^2^ and the alamarBlue® (Invitrogen, United Kingdom) assay was carried out at day 4 and day 10 of culture, according to the manufacturer’s protocol. Briefly, samples were washed with HBSS and left to incubate in HBSS containing 10% alamarBlue® for 3 h at 37°C in a humidified atmosphere of 5% CO_2_. After incubation, 100 μl of the alamarBlue® solution were transferred into a 96-well plate. Absorbance readings were measured at 550 nm excitation and 595 nm emission with a Varioskan Flash Spectral scanning multimode reader (ThermoFisher Scientific, United Kingdom). Metabolic activity was expressed in terms of % of reduced alamarBlue™ dye normalised to the DNA quantity (ng/mL) obtained from the Quant-iT™ PicoGreen® dsDNA assay. Each value was normalised to the value of α-MEM -MMC group.

### 2.8 Electrophoresis analysis

To assess collagen type I deposition sodium dodecyl sulphate-polyacrylamide gel electrophoresis (SDS-PAGE) was performed at p4, at a seeding density of 25,000 cells/cm^2^, at day 4 and at day 10 of culture, as has been described previously (Capella-Monsonis et al., 2018). Briefly, at each time point media were aspired and cell layers were washed with HBSS. Subsequently, cell layers were digested with porcine gastric mucosa pepsin at a final concentration of 0.1 mg/mL in 0.05 M acetic acid (Fischer Scientific, Ireland) and incubated for 2 h at 37°C with gentle shaking. After digestion, cell layers were scraped and neutralised with 0.1 N sodium hydroxide. For electrophoresis, sample buffer (SDS, 1.25 M Tris HCl, glycerol, bromophenol blue) was added to the samples. Cell layers were analysed by SDS-PAGE under non-reducing conditions with a Mini-Protean® three electrophoresis system (Bio-Rad Laboratories, United Kingdom). Bovine collagen type I (125 μg/mL, Symatese Biomateriaux, France) was used as control on every gel. Protein bands were stained with the SilverQuest™ kit (Invitrogen) according to the manufacturer’s protocol. The gels were imaged with a HP PrecisionScan Pro scanner (HP, United Kingdom). Densitometric analysis of the α1 and α2 bands was performed with ImageJ software (NIH).

### 2.9 Immunocytochemistry analysis

At p4, cells were seeded in 48-well plates (Sarstedt, Ireland) at a density of 25,000 cells/cm^2^. At each time point cells were washed with PBS and fixed in 4% paraformaldehyde for 15 min at room temperature. Cells were washed with PBS and then non-specific sites were blocked with 3% bovine serum albumin for 30 min. Afterwards, cells were incubated overnight at 4°C with primary antibodies against collagen type I (Supplementary Table S2). Cells washed 3 times with PBS and incubated for 45 min at room temperature with the secondary antibody (Supplementary Table S2). Nuclei were counterstained with Hoechst 33342 Fluorescent Stain (ThermoFisher Scientific, United Kingdom). Images were acquired using an Olympus IX-81 inverted fluorescence microscope (Olympus Corporation, Japan) and relative fluorescence intensity was analysed with ImageJ software (NIH, United States).

### 2.10 Trilineage differentiation analysis

For all differentiation experiments (Supplementary Table S3 provides the groups), cells at p4 were subjected to MesenCult™ Adipogenic Differentiation Kit (STEMCELL Technologies, United Kingdom), MesenCult™ Osteogenic Differentiation Kit (STEMCELL Technologies, United Kingdom) and MesenCult™-ACF Chondrogenic Differentiation Kit (STEMCELL Technologies, United Kingdom), according to the manufacturer’s protocols. For adipogenic and osteogenic differentiation cells were seeded in 48-well plates at an initial density of 25,000 cells/cm^2^, differentiation was commenced when cells were approximately 90%–98% confluent, and media was changed every 3 days. For chondrogenic differentiation a 3D pellet culture system was used with 500,000 cells/pellet. For MMC conditions, the same as during stem cell expansion Fc cocktail was used.

#### 2.10.1 Adipogenic differentiation, oil red O staining and quantification of uptake

After 14 days of adipogenic differentiation, phase contrast images were captured using an inverted brightfield microscope (Leica Microsystem, Germany). Cells were then fixed for 20 min with 4% paraformaldehyde, stained for 15 min with oil red O solution (oil red O 0.5% in isopropanol, diluted 3:2 in deionised water) at room temperature and images were acquired using an inverted microscope (Leica Microsystems, Germany). For semi-quantitative analysis of oil red O staining, the dye was extracted with 100% isopropanol, the solution was centrifuged at 500 *g* for 2 min, and absorbance was measured at 520 nm using a Varioskan Flash plate reader (ThermoFisher Scientific).

#### 2.10.2 Osteogenic differentiation, alizarin red staining and quantification of uptake

After 14 days of osteogenic differentiation, phase contrast images were captured using an inverted brightfield microscope (Leica Microsystem, Germany). Cells were then fixed with ice-cold methanol for 20 min, stained with 2% alizarin red solution in deionised water for 15 min and washed three times with deionised water. Brightfield images were acquired using an inverted microscope (Leica Microsystems, Germany). Semi-quantitative analysis of alizarin red staining was performed by dissolving the bound stain with 10% acetic acid. Samples were collected using a cell scraper and heated to 85°C for 10 min. Subsequently, 10% solution of ammonium hydroxide was used to adjust the pH to 4.5, and absorbance at 405 nm was read using a micro-plate reader (Varioskan Flash, ThermoFisher Scientific, Ireland).

#### 2.10.3 Chondrogenic differentiation and Alcian Blue staining

For pellet culture, cells were directly resuspended in chondrogenic differentiation medium, 0.5 mL of the cell suspension was added to each 15 mL polypropylene tube and centrifuged at 300 *g* for 10 min. Cells were incubated at 37°C in a humidified atmosphere of 5% CO₂. On day 3, 0.5 mL chondrogenic media was added to reach a final volume of 1 mL and subsequently media was changed every 3 days. After 21 days of differentiation, pellets were fixed with 4% paraformaldehyde, cryoprotected with 15% and 30% solutions of sucrose in one x PBS (w/vol), cryo-embedded and cryo-sectioned (5 μm) with a Leica Cryostat (Leica Biosystems, Germany). To assess the presence of proteoglycans sections were stained with Alcian Blue 8GX solution (Sigma-Aldrich 66011) for 30 min at room temperature and counterstained with Nuclear fast red (Nuclear fast red–aluminium sulphate solution 0.1%, Merck Millipore, 1001210500) for 1 min at room temperature. Slides were dehydrated in 100% ethanol, xylene and mounted. Brightfield images were acquired using an inverted microscope (Leica Microsystems, Germany).

### 2.11 Statistical analysis

For both donors (N = 2 biological replicates), all experiments were conducted in three technical replicates (*n* = 3). Due to limitations in cell numbers, flow cytometry assays were performed one time (*n* = 1 technical replicate) for each donor (N = 2). Data were processed using MINITAB® version 17 (Minitab Inc., United States) and reported as mean ± standard deviation. One-way analysis of variance (ANOVA) was used for multiple comparisons and Tukey’s *post hoc* test was used for pairwise comparisons when the group distributions were normal (Anderson-Darling normality test) and the variances of populations were equal (Bonett’s test and Levene’s test). When either or both assumptions were violated, non-parametric analysis was conducted using Kruskal–Wallis test for multiple comparisons and Mann-Whitney test for pairwise comparisons. Results were considered statistically significant for *p* < 0.05.

## 3 Results

### 3.1 Cell immunophenotype

Flow cytometry analysis at p0 (Supplementary Figures S1-S2; Supplementary Tables S4-S5) revealed that hBMSCs of both donors that were isolated in ACF, were positive for CD90, CD73, CD44, CD105 and CD146 and negative for the haematopoietic markers CD31 and CD45. However, cells of donor two exhibited an elevated population of CD45^+^ cells at p0.

Flow cytometry analysis at p4 (Supplementary Figures S3-S4; Supplementary Tables S4-S5) revealed that hBMSCs of both donors continued to express CD90, CD44, CD73 and CD105 at high levels in most culture conditions. Reduced levels of CD105 were detected for donor one in FBS -MMC, XF -MMC and ACF + MMC, and for donor two in FBS -MMC, FBS + MMC, ACF -MMC and ACF + MMC. The addition of MMC retained high CD105 values in both FBS and XF groups. In contrast, ACF + MMC showed lower CD105 values compared to ACF-MMC. While cells of both donors showed relatively high CD146 expression in p0 (> 70% for donor 1, >50% for donor 2), CD146 dramatically decreased in donor one cells in FBS -MMC (>10%), and in donor two cells in FBS–MMC, FBS + MMC (>15%). With respect to negative markers at p4, only donor one cells in XF + MMC and ACF -MMC, and donor two cells in XF -MMC and XF + MMC increased CD31 expression over 40%. While donor one cells did not upregulate CD45 at p4 (> 5%), donor two cells increased CD45 expression in most culture conditions over 40% (except ACF -MMC).

### 3.2 Cell morphology analysis

For both donors, qualitative cell morphology analysis (Supplementary Figure S5A-B) revealed that cell morphology was not affected as a function of ACF and XF media formulations and MMC supplementation, whilst cells expanded with serum-containing media adopted a slightly rounder, cuboidal shape at p4.

### 3.3 Cell viability analysis

For cells isolated from both donors, no significant differences were observed in cell viability (Supplementary Figure S6A-D) as a function of media formulation and MMC supplementation. Percentages of live cells were ≥90% for all experimental conditions and for cells of both donors.

### 3.4 DNA concentration analysis

DNA concentration analysis revealed that cells from the two donors exhibited different proliferation behaviours in the various media (Supplementary Figure S7A-B). For donor 1 (Supplementary Figure S7A), no statistically significant differences were evident between groups after 4 days of culture. At day 10, serum-containing conditions showed significantly lower (*p* < 0.05) DNA concentration when compared to all XF- and ACF conditions, regardless of MMC.

DNA concentration analysis for donor 2 (Supplementary Figure S7B) revealed that the FBS + MMC induced significantly (*p* < 0.05) highest DNA concentration among all groups at day 4At day 10, DNA concentration in XF -MMC, XF + MMC, and ACF -MMC was significantly (*p* < 0.05) higher than DNA concentration in all other conditions. Overall, serum-containing conditions showed lower proliferation rates than XF and ACF conditions.

### 3.5 Cell metabolic activity analysis

Cell metabolic activity analysis revealed that cells from the two donors exhibited different metabolic activity in the various media (Supplementary Figure S7C-D). For donor 1 (Supplementary Figure S7C), cell metabolic activity analysis at day 4 revealed that the FBS + MMC and XF + MMC induced significantly (*p* < 0.05) higher metabolic activity than the XF -MMC, ACF -MMC and ACF + MMC. At day 10, cell metabolic activity in FBS was significantly (*p* < 0.001) higher than cell metabolic activity in ACF and XF.

For donor 2 (Supplementary Figure S7D), cell metabolic activity in FBS -MMC, was significantly (p < 0.05) higher than cell metabolic activity in all other conditions at day 4. At day 10, cell metabolic activity in FBS -MMC was significantly higher than cell metabolic activity in all other conditions (*p* < 0.05). Overall, a decrease in metabolic activity from day 4 to day 10 was observed for cells from both donors in all culture conditions.

### 3.6 Electrophoresis analysis

**FIGURE 2:**
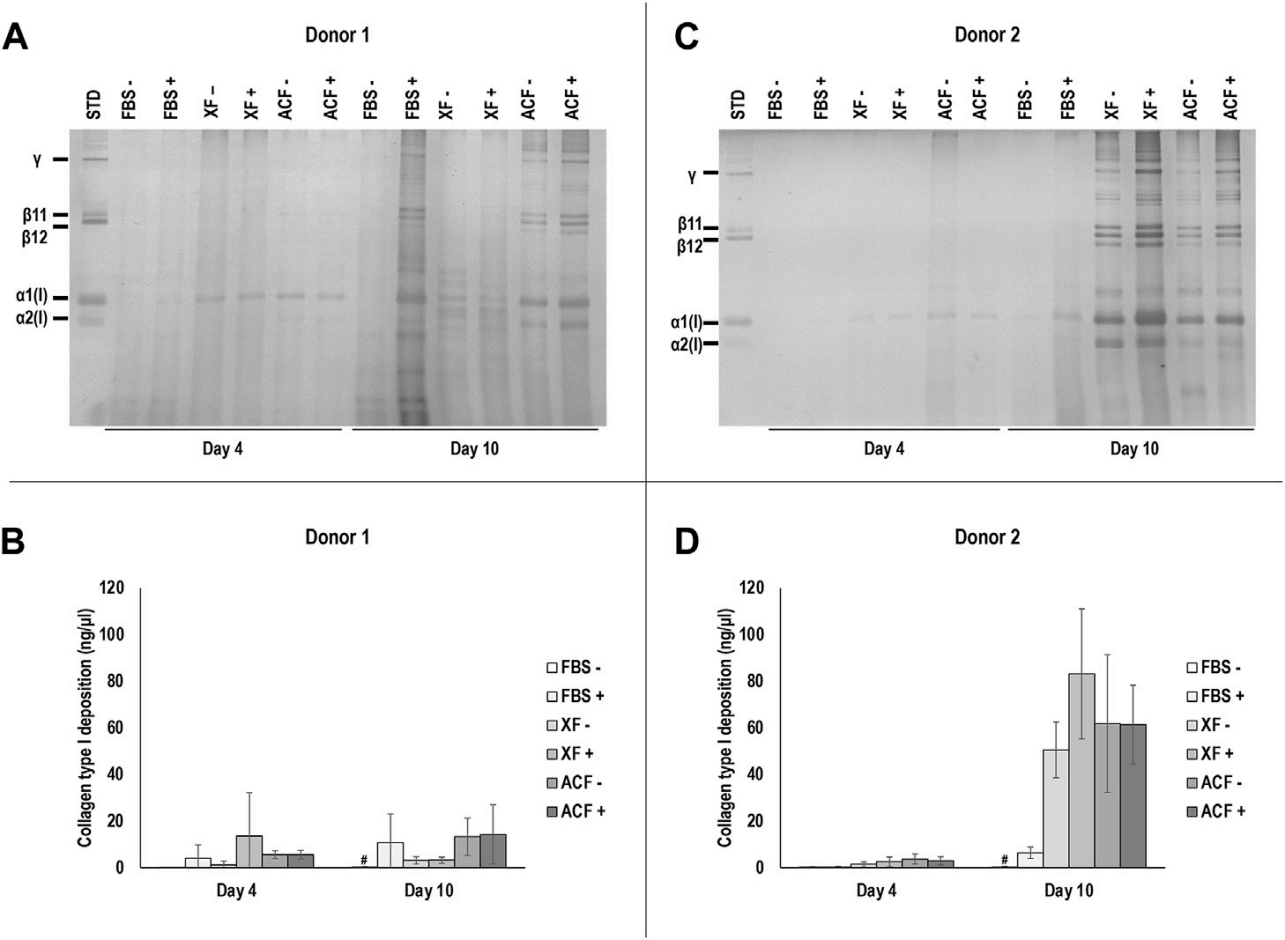
SDS-PAGE and corresponding densitometric analysis of hBMSCs of donor 1 **(A, B)** and 2 **(C, D)** at p4 after 4 and 10 days of culture, expanded with or without MMC in ACF, XF and FBS containing media. Experiments for cells of each donor were performed in three technical replicates. # indicates the lowest statistically significant value (*p* < 0.05) at a given time point.

SDS-PAGE and corresponding densitometric analysis (Figures 2A–D) revealed similar collagen deposition profiles for cells from both donors.

For both donors at day 4, no significant (*p* > 0.05) differences in collagen deposition was observed among all conditions. For both donors at day 10, FBS -MMC showed significantly lower (*p* < 0.05) collagen deposition compared to all other groups, regardless of MMC. In all ACF and XF conditions, the presence of MMC did not significantly (*p* > 0.05) affect collagen deposition. No protein bands were detected in silver-stained SDS-PAGE when attachment solutions only were analysed (Supplementary Figure S11A). In summary, MMC increased collagen type I deposition in FBS (both donors) and in XF (donor 2) media at day 10, and XF and ACF conditions induced overall higher collagen deposition in donor two cells compared to donor one cells on day 10.

### 3.7 Immunocytochemistry analysis

Immunocytochemistry (ICC) for collagen type I (Supplementary Figure S8A–C) and complementary relative fluorescence intensity analysis (Supplementary Figure S8B–D) show similar collagen type I deposition profiles for cells of both donors. While MMC did not significantly (*p* > 0.05) increase collagen type I deposition in all ACF and XF conditions for donor 1 (Supplementary Figure S8A-B), MMC significantly (*p* < 0.05) increased collagen type I deposition in FBS at day 4 and day 10. Overall, collagen type I deposition did not significantly (*p* > 0.05) increase between day 4 and day 10 in none of the groups.

For donor 2 (Supplementary Figure S8C-D), the ACF with MMC at day 4 resulted in the highest (*p* < 0.05) collagen type I deposition across all groups and time points. At day 10, collagen type I deposition was significantly (*p* < 0.05) lowest in FBS -MMC. ICC for collagen type I and fibronectin of attachment solutions only did not show any positive staining (Supplementary Figure S11B). In summary, XF and ACF conditions induced overall higher collagen type I deposition in donor two cells compared to donor one cells on day 10.

### 3.8 Trilineage differentiation analysis

**FIGURE 3:**
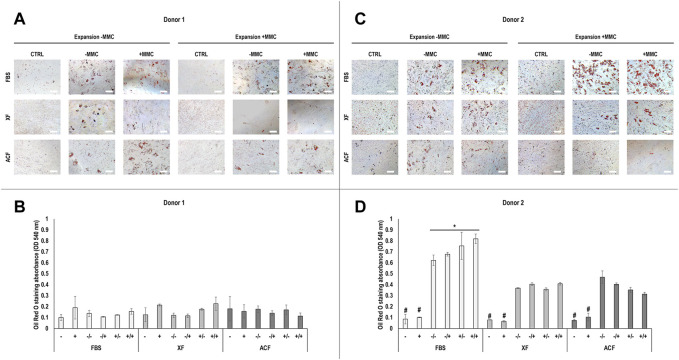
Oil Red O staining and corresponding semi-quantitative absorbance analysis of hBMSCs of donor 1 **(A, B)** and 2 **(C, D)**, expanded with or without MMC in ACF, XF and FBS containing media, and differentiated with adipogenic induction media with or without MMC supplementation at p4. Experiments were performed in three technical replicates. # indicates the lowest statistically significant value (*p* < 0.05) at a given time point. * indicates the highest statistically significant value (*p* < 0.05) at a given time point. Scale bars: 100 μm.

Phase contrast images (Supplementary Figure S9A-B) and Oil Red O staining and corresponding absorbance analysis (Figures 3A–D) of hBMSCs expanded without (−) or with (+) MMC in the respective expansion media and differentiated with adipogenic induction media without (−) or with (+) MMC at p4 revealed differences with respect to donor, media, and MMC. For donor 1 (Figure 3A), FBS condition showed higher lipid droplet accumulation compared to ACF and XF conditions when analysed qualitatively, regardless of whether MMC was used during expansion or differentiation. However, these differences were not statistically significant (*p* > 0.05) (Figure 3B). No lipid droplet accumulation was detected in XF +/+ conditions. For donor 2 (Figures 3C,D), FBS conditions induced significantly (*p* < 0.05) higher lipid droplet accumulation compared to ACF and XF conditions, regardless of whether MMC was used during expansion or differentiation. Qualitatively (Figure 3C), FBS +/+ induced higher lipid deposition than FBS expanded without MMC, regardless of the presence of MMC in the differentiation media (FBS −/−, FBS −/+). Overall, cells of both donors expanded in FBS conditions showed higher adipogenic differentiation potential than all ACF and XF groups.

**FIGURE 4:**
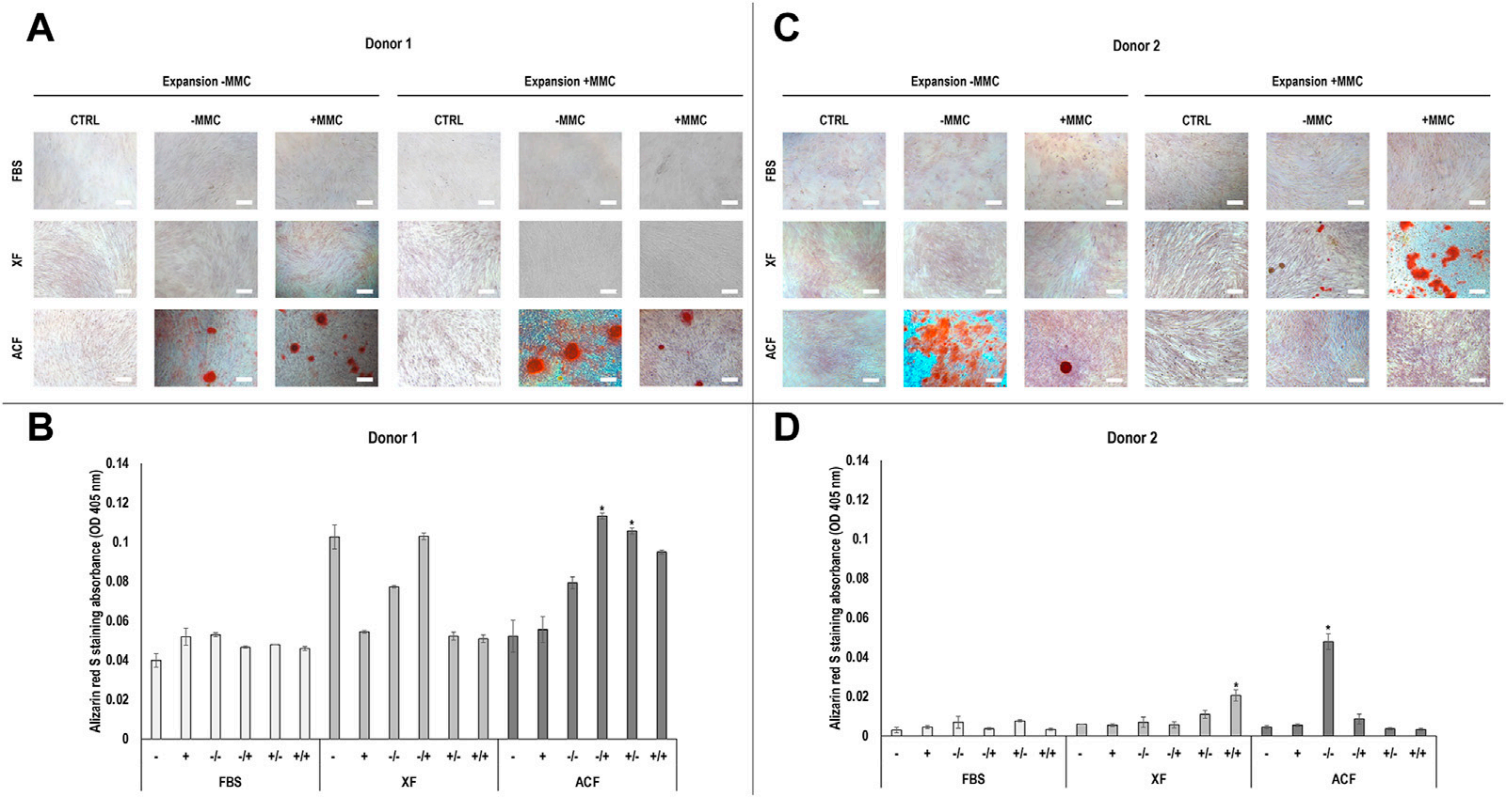
Alizarin red staining and corresponding semi-quantitative absorbance analysis of hBMSCs of donor 1 **(A, B)** and 2 **(C, D)**, expanded with or without MMC in ACF, XF and FBS containing media, and differentiated with osteogenic induction media with or without MMC supplementation at p4. Experiments were performed in three technical replicates. # indicates the lowest statistically significant value (*p* < 0.05) at a given time point. * indicates the highest statistically significant value (*p* < 0.05) at a given time point. Scale bars: 100 μm.

Phase contrast images (Supplementary Figure S10A-B) and alizarin red staining and corresponding absorbance analysis (Figures 4A–D) of hBMSCs expanded without (−) or with (+) MMC in the respective expansion media and differentiated with osteogenic induction media without (−) or with (+) MMC in p4 revealed differences with respect to donor, media, and MMC. For both donors (Figures 4A–D), no calcium deposition was detected in FBS groups, regardless of whether MMC was used during expansion or differentiation. For donor 1 (Figures 4A,B), no calcium deposition was detected in XF groups, regardless of whether MMC was used during expansion or differentiation. Calcium deposition was detected in all ACF groups; the ACF −/+ group induced significantly (*p* < 0.05) higher calcium deposition than the ACF +/+ and ACF −/− groups. For donor 2 (Figures 4C,D), no calcium deposition was detected when hBMSCs were expanded in XF without MMC, independently on whether MMC was used during differentiation. When hBMSCs were expanded in XF with MMC, the XF +/+ induced significantly (*p* < 0.05) higher calcium deposition than the XF +/−. ACF groups showed calcium deposition only when hBMSCs were expanded without MMC and the ACF −/− induced significantly (p < 0.05) higher calcium deposition than ACF −/+ and all other ACF, XF and FBS conditions. Overall, cells of both donors showed high osteogenic potential when expanded in ACF -MMC conditions.

**FIGURE 5:**
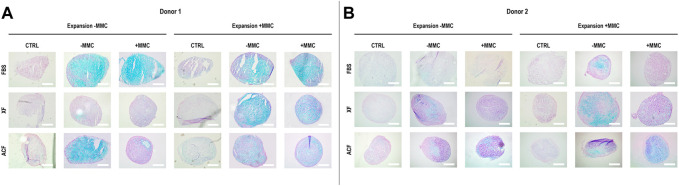
Alcian blue staining for sulphated proteoglycans of hBMSCs of donor 1 **(A)** and 2 **(B)**, expanded with or without MMC in ACF, XF and FBS containing media, and differentiated in pellet culture with chondrogenic differentiation media with or without MMC supplementation at p4. Experiments were performed in three technical replicates.

Qualitative analysis of Alcian blue staining (Figures 5A,B) of hBMSCs expanded without (−) or with (+) MMC in the respective expansion media and differentiated with chondrogenic induction media without (−) or with (+) MMC in p4 for donor one revealed a proteoglycan-rich ECM in all FBS conditions, regardless of whether MMC was used during expansion or differentiation (Figure 5A). For ACF and XF conditions, only the XF ± and XF +/+ groups resulted in proteoglycan-rich ECM. For the ACF groups, proteoglycan-rich ECM was detected for ACF −/− and little to no chondrogenic differentiation was detected for ACF −/+. When hBMSCs were expanded in ACF with MMC, a proteoglycan-rich ECM was detected independently on whether MMC was used during differentiation.

For donor 2, qualitative analysis of Alcian blue staining (Figure 5B) revealed a proteoglycan-rich ECM for all conditions, except for FBS −/+, FBS +/+ and XF −/+. Overall, donor two chondrogenic pellets showed a relatively lower proteoglycan content when compared to donor one groups. Overall, donor one cells expanded with MMC showed a higher proteoglycan deposition and chondrocyte-like cells with lacunae-formation across all groups compared to donor two cells.

## 4 Discussion

Animal-derived cell culture media and absence of native ECM are associated with hBMSC phenotypic drift and loss of their therapeutic potential. To alleviate these issues in the developmental cycle of stem cell-based medicines, the use of either ACF or XF media formulations and MMC have been proposed. Interestingly, their combined effect has only been assessed once in human adipose-derived mesenchymal stromal cell cultures (Patrikoski et al., 2017). Herein, we ventured to assess whether ACF or XF media formulations supplemented with MMC (either/or during expansion and either/or during differentiation) can more effectively control hBMSCs fate than FBS-based media formulations supplemented with MMC (either/or during expansion and either/or during differentiation). To induce artificial polydispersity for maximum excluded volume effect, and consequently increased ECM deposition by cells, we used a previously optimised (Gaspar et al., 2019) MMC cocktail with different molecular weights of the same crowder (Ficoll®).

### 4.1 Surface marker analysis

While hBMSCs of both donors exhibited a surface marker profile according to the International Society for Cellular Therapy criteria (Dominici et al., 2006) at p0, donor two cells showed elevated levels of the haematopoietic marker CD45. It was previously reported that freshly isolated hBMSCs expressed CD45, while in cultured MSCs and cells at later passages CD45 was downregulated. These cells were capable to differentiate into osteochondroblastic cells, adipocytes and stromacytes. These results are in agreement with our results for donor two hBMSCs expanded with ACF -MMC. Even though cells mildly expressed CD45 at p0, the hematopoietic marker was almost absent at p4 when kept in ACF media without MMC during expansion (Deschaseaux et al., 2003). At p4, a decrease in CD105 and CD146 and an increase in CD31 and CD45 levels was observed at p4 in some conditions. It is worth noting that one study has shown the expression of CD146 in hBMSCs to decrease with increasing passage number (from 95.1% in p3 to 49.7% in p8) (Yang et al., 2018b), whilst other studies have shown the expression of CD105 to increase in hADSCs in higher [e.g., p3 (Yoshimura et al., 2006), p4 (Varma et al., 2007)] passages. The different expression patterns for CD105 and CD146 could therefore be influenced by donor, culture media and MMC. However, it needs to be noted that hADSCs have a different surface marker profile than hBMSCs. CD31 increased to highest values in donor two cells expanded in XF media, regardless of the presence of MMC. While CD45 remained low in all donor one conditions, for donor 2, it remained low only for ACF -MMC and increased to highest values in both XF media conditions. These results clearly suggest donor variability and effects of culture media on stem cell phenotype. It is also worth noting that a study has shown high expression of CD45 in freshly isolated and commercially available hBMSCs from different donors throughout the expansion period (Okolicsanyi et al., 2015). Furthermore, increased expression levels of CD31 and CD45 in later passage adipose-derived mesenchymal stromal cells were associated with presence of endothelial and haematopoietic cells (Wan Safwani et al., 2011). All these data indicate that attention should be paid when surface markers are used to characterise MSCs, as variations in their expression is well documented in the literature (Mafi et al., 2011). Among both donors and all conditions assessed herein, cells expanded in ACF media showed overall high surface expression of positive MSC markers and relatively low expression of CD31 and CD45, indicative of phenotype maintenance, despite a relatively low CD105 expression in donor two cells. Across all media, donor one cells better maintained their stromal phenotype compared to donor two cells. This is in accordance with previous publications with hBMSCs (Russell et al., 2010; Siegel et al., 2013) and can be attributed to donor variability. To be consistent with other experiments at p4, immunophenotyping at p4 was performed after 10 days of culture when cells have had grown to confluency, which may have affected their surface marker profile.

### 4.2 Basic cellular function analysis

Cell viability of both donors was not negatively affected by media conditions. With respect to DNA concentration, the only clear trend observed among both donors was that in the absence of MMC, the ACF and XF media formulations induced significantly higher DNA concentration than their FBS counterparts. This increased DNA concentration in ACF and XF media formulations can be attributed to the various additives used to replace animal sera. For example, a previous study showed that adipose-derived mesenchymal stromal cells cultured in serum-free media had a higher population doubling time compared to adipose-derived mesenchymal stromal cells cultured with FBS (Lee et al., 2022). Another study showed that hBMSCs cultured in serum-free media had higher proliferation rates compared to cells cultured in serum-containing media (Chase et al., 2010). With respect to metabolic activity, among both donors, the FBS without MMC almost across the board induced the highest metabolic activity. This is in agreement with a previous publication, in which adipose-derived mesenchymal stromal cells cultured with FBS had significantly higher metabolic activity compared to cells cultured in human serum or xeno-free conditions (Patrikoski et al., 2017). Further, alamarBlue® assay is based on the reduction of resazurin to resorufin by mitochondrial enzymes, like NADPH dehydrogenase (O'Brien et al., 2000). It has been demonstrated that culture expansion with serum leads to a progressive decline of intracellular NAD + levels and increase in NADH levels, which together change the redox cycle balance in high passages of hMSCs, connecting mitochondrial fitness with replicative senescence in hMSCs (Patrikoski et al., 2017; Yuan et al., 2020). Thus, the relatively higher metabolic activity of hBMSCs cultured with FBS could indicate senescence or phenotype loss. No clear trend with respect to MMC was observed for DNA concentration and metabolic activity. One should note that Ficoll™ is used extensively in stem cell purification (Jaatinen and Laine, 2007; Kawasaki-Oyama et al., 2008; Al Battah et al., 2011; Najar et al., 2014; Kakabadze et al., 2019) and as MMC agent (Zeiger et al., 2012; Rashid et al., 2014; Gaspar et al., 2019), and to-date, no negative data have been reported. Overall, differences in DNA concentration and metabolic activity between donors could also be a result of the differences observed in surface marker expression of positive and negative MSC markers. In addition, contact inhibition may have affected proliferation rates in confluent cultures on day 10.

### 4.3 Collagen deposition analysis

SDS-PAGE analysis revealed that MMC increased collagen type I deposition in FBS (both donors) and in XF (donor 2) media formulations at day 10, but not at day 4. Ficoll™ cocktails have been shown repeatedly to enhance and accelerate ECM deposition in both permanently differentiated (Kumar et al., 2015b) and stem cell (Cigognini et al., 2016) cultures. Further, Ficoll™ cocktails are known to require longer periods of time to enhance ECM deposition than natural [such as carrageenan (Satyam et al., 2014; Gaspar et al., 2019)] or synthetic [such as dextran sulphate and polyvinylpyrrolidone (Chen et al., 2009; Rashid et al., 2014)] macromolecules. It is worth noting that FBS containing media induced significantly lower collagen synthesis and/or deposition (as assessed *via* SDS-PAGE) than the XF and ACF media formulations. We attribute these observations to the presence of matrix metalloproteinases that degrade collagen in FBS (Satyam et al., 2014; Kumar et al., 2015a) and the presence of growth factors that allow for cell attachment and growth in XF and ACF media formulations (Chase et al., 2010). Interestingly, the XF with MMC induced the highest collagen deposition, as judged by SDS-PAGE. We attribute this to the synergistic effect of the contained growth factors with MMC. Indeed, MMC combined with growth factor supplementation resulted in amplified (over cells with growth factors alone and cells with MMC alone) collagen deposition, as the growth factors enhanced collagen synthesis and MMC enhanced collagen deposition (Tsiapalis et al., 2021). Following the same reasoning, one would have expected the ACF with MMC to induce higher collagen deposition than the ACF alone, which was not the case, as judged by SDS-PAGE. For this, we believe that the concentration of the Ficoll™ cocktail used was not sufficient to effectively exclude volume and an optimisation study should be conducted.

The immunofluorescence analysis only for donor one at day 10 validated the SDS-PAGE results with respect to higher ECM deposition when ACF and XF, as opposed to FBS, were used and higher ECM deposition when MMC was used in FBS cultures. MMC also effectively increased ECM deposition in ACF at both time points with donor two cells. The MMC data are in clear contradiction to previous studies (Gaspar et al., 2019), where increased ECM deposition has been comprehensively demonstrated in the presence of Ficoll™. The only logical explanation is the sensitivity of the assay, as opposed to the sensitivity of silver-stained gels than can reach 0.05–0.2 ng (Jin et al., 2004).

### 4.4 Adipogenic differentiation analysis

Overall, hBMSCs isolated from both donors showed sufficient potential to differentiate into the adipogenic lineage. Even though CD31 and CD45 expression was relatively high in donor two cells at p4, previous studies reported that CD45-positive adipose-derived mesenchymal stromal cells possessed adipogenic potential *in vitro* (Yu et al., 2010). Qualitatively, hBMSCs expanded in serum-containing media showed higher adipogenic differentiation compared to ACF/XF conditions, which was further enhanced by MMC during expansion. An overall low lipid droplet formation in donor one cells resulted in no statistically significant differences in Oil red O uptake. Relatively high values in negative controls (no differentiation media) are due to higher cell proliferation and entrapment of Oil Red O in the deposited ECM. These results are in agreement with previous studies, where high serum concentrations (10%, 20% FBS) enhanced adipogenic differentiation of MSCs by activation of the MEK/ERK signalling pathway, ultimately promoting PPARγ expression and phosphorylation, compared to low serum culture (2% FBS) (Wu et al., 2010). Further, MMC (Ficoll^™^) was reported to increase adipogenic differentiation of hBMSCs by promoting a pro-adipogenic microenvironment (Levengood and Zhang, 2014); to facilitate brown adipocyte differentiation through MMC-enhanced collagen type IV formation in adult hBMSCs (Lee et al., 2016) and to promote the differentiation of adipocytes (Chen et al., 2023).

### 4.5 Osteogenic differentiation analysis

As no calcium deposition was detected in FBS conditions for both donors, independent of MMC, cells possibly committed towards the adipogenic lineage during expansion, as evidenced by high adipogenic differentiation capacity. In XF media, only donor two cells deposited abundant calcium nodules when expanded with +MMC. Interestingly, these groups showed the highest expression of CD31 and CD45. Previous studies reported that CD45-positive adipose-derived mesenchymal stromal cells possessed osteogenic potential *in vitro* (Yu et al., 2010). For donor two cells expanded with MMC, MMC during differentiation significantly increased calcium nodule formation. Studies showed carrageenan (Cigognini et al., 2016; Graceffa and Zeugolis, 2019) and dextran sulphate 500 kDa (Assunção et al., 2020) enhanced osteogenic differentiation of BMSCs, in serum-containing culture. Other studies showed osteogenic potential of a Ficoll™ 70 kDa and Ficoll™ 400 kDa cocktail (Patrikoski et al., 2017) and sulphated seaweed polysaccharides (De Pieri et al., 2020) in serum-containing human adipose-derived mesenchymal stromal cell cultures. CD146 is considered one of the most appropriate stemness markers, as it is universally detected in MSCs isolated from various tissues and associated with higher multipotency (Crisan et al., 2008; Lv et al., 2014). CD146 was reported to be heterogeneous on a subset of BMSCs (Bühring et al., 2009). Osteoprogenitor cells have been reported to be highly positive for CD146 (Sacchetti et al., 2007) and thus a reduction in CD146 over passages may compromise their osteogenic potential (Yang et al., 2018b). Interestingly, cells of both donors expanded with ACF medium showed sufficient osteogenic potential, except for donor two cells when MMC was present during differentiation. Cells of both donors expanded with ACF/XF media showed higher expression of CD146, compared to FBS, indicating that ACF/XF media supported multipotency, and therefore possibly higher osteogenic potential. Differences in osteogenic potential observed in our study can further be attributed to donor variability and differences in media formulations. A previous study reported variability in osteogenic potential of MSCs from 19 different donors, irrespective of age, gender, and source of isolation, which was attributed to cellular heterogeneity among donors (Siddappa et al., 2007).

### 4.6 Chondrogenic differentiation analysis

A lower chondrogenic and osteogenic potential of donor two cells corroborates with relatively high expression of CD31 and CD45, indicating phenotype loss. Even though donor one cells show decreased CD105 expression at p4, previous studies showed that chondrogenic differentiation potential of BMSCs was not linked to CD105 levels (Cleary et al., 2016). Across both donors, MMC during expansion increased proteoglycan-deposition in hBMSCs. MMC has previously been shown to increase chondrogenic differentiation in hBMSCs (Cigognini et al., 2016), and human adipose-derived mesenchymal stromal cells (De Pieri et al., 2020). CD146+ MSCs have been associated with enhanced chondrogenesis and greater therapeutic potential for collagen-induced arthritis (Hagmann et al., 2014; Wu et al., 2016; Li et al., 2019). Here, higher CD146 expression in donor one compared to donor two cells is reflected in its chondrogenic potential. Interestingly, high deposition of sulphated proteoglycans in MMC groups coincides with higher expression of CD146 in p4. Both supportive and inhibitory effects of MMC on human adipose-derived mesenchymal stromal cell culture have been shown to be culture condition dependent (Mittal et al., 2015; Patrikoski et al., 2017).

### 4.7 Limitations and future perspectives

Due to unknown composition of ACF/XF media, some results could not be fully explained. Possible inhibitory interactions of Ficoll^™^ with certain media components cannot be ruled out. Changing media formulations between cell isolation and expansion may have affected cell phenotype and differentiation capacity. To limit the introduction of unknown variables to this study cells of different experimental groups were isolated using the same isolation media and protocol and differentiated with the same differentiation media and protocols. Therefore, the experimental groups only differed in media and protocols for expansion. Future studies could use compatible media and protocols for isolation, expansion, and differentiation derived from the same company, for each experimental group. An additional positive control group per donor should be added, in which hBMSCs are isolated and expanded with FBS-containing media, and trilineage experiments for this group are performed with established in-house protocols. In addition, future studies should use a higher number of donors to account for donor variability, and calculate cumulative population doubling (cPD) levels instead of passage number to appropriately track cellular aging across different conditions.

## 5 Conclusion

Contemporary tissue engineered therapies require development of clinically relevant cell culture media that enhance and accelerate ECM deposition to reduce manufacturing costs, whilst maintaining cellular phenotype and function. In this context, herein we studied the influence of culture media (FBS, ACF, XF) without/with MMC in hBMSC (from two different donors) fate. Our data indicate that cell behaviour depends on donor and media formulation. Investigators should carefully select culture conditions for cell expansion and differentiation and consider potential cross-reactions between media supplements (e.g., macromolecular crowding molecules) and base media (e.g., chemically defined media).

## Data Availability

The original contributions presented in the study are included in the article/Supplementary Material, further inquiries can be directed to the corresponding author.

## References

[B1] Al Battah F. De Kock J. Ramboer E. Heymans A. Vanhaecke T. Rogiers V. (2011). Evaluation of the multipotent character of human adipose tissue-derived stem cells isolated by Ficoll gradient centrifugation and red blood cell lysis treatment. Vitro 25, 1224–1230. 10.1016/j.tiv.2011.05.024 21645610

[B2] Assunção M. Wong C. W. Richardson J. J. Tsang R. Beyer S. Raghunath M. (2020). Macromolecular dextran sulfate facilitates extracellular matrix deposition by electrostatic interaction independent from a macromolecular crowding effect. Mater Sci. Eng. C Mater Biol. Appl. 106, 110280. 10.1016/j.msec.2019.110280 31753359

[B3] Bara J. J. Richards R. G. Alini M. Stoddart M. J. (2014). Concise review: Bone marrow-derived mesenchymal stem cells change phenotype following *in vitro* culture: Implications for basic research and the clinic. Stem Cells 32, 1713–1723. 10.1002/stem.1649 24449458

[B5] Bühring H. J. Treml S. Cerabona F. de Zwart P. Kanz L. Sobiesiak M. (2009). Phenotypic characterization of distinct human bone marrow-derived MSC subsets. Ann. N. Y. Acad. Sci. 1176, 124–134. 10.1111/j.1749-6632.2009.04564.x 19796240

[B6] Capella-Monsonis H. Coentro J. Q. Graceffa V. Wu Z. Zeugolis D. I. (2018). An experimental toolbox for characterization of mammalian collagen type I in biological specimens. Nat. Protoc. 13, 507–529. 10.1038/nprot.2017.117 29446773

[B7] Chase L. G. Lakshmipathy U. Solchaga L. A. Rao M. S. Vemuri M. C. (2010). A novel serum-free medium for the expansion of human mesenchymal stem cells. Stem Cell Res. Ther. 1, 8. 10.1186/scrt8 20504289 PMC3226302

[B8] Chase L. G. Yang S. Zachar V. Yang Z. Lakshmipathy U. Bradford J. (2012). Development and characterization of a clinically compliant xeno-free culture medium in good manufacturing practice for human multipotent mesenchymal stem cells. Stem Cells Transl. Med. 1, 750–758. 10.5966/sctm.2012-0072 23197667 PMC3659659

[B9] Chen C. Z. Peng Y. X. Wang Z. B. Fish P. V. Kaar J. L. Koepsel R. R. (2009). The Scar-in-a-Jar: Studying potential antifibrotic compounds from the epigenetic to extracellular level in a single well. Br. J. Pharmacol. 158, 1196–1209. 10.1111/j.1476-5381.2009.00387.x 19785660 PMC2782330

[B10] Chen H. Yan X. Sun A. Zhang L. Zhang J. Yan Y. (2023). Adipose extracellular matrix deposition is an indicator of obesity and metabolic disorders. J. Nutr. Biochem. 111, 109159. 10.1016/j.jnutbio.2022.109159 36162565

[B11] Cheng C. W. Solorio L. D. Alsberg E. (2014). Decellularized tissue and cell-derived extracellular matrices as scaffolds for orthopaedic tissue engineering. Biotechnol. Adv. 32, 462–484. 10.1016/j.biotechadv.2013.12.012 24417915 PMC3959761

[B12] Cigognini D. Gaspar D. Kumar P. Satyam A. Alagesan S. Sanz-Nogues C. (2016). Macromolecular crowding meets oxygen tension in human mesenchymal stem cell culture - a step closer to physiologically relevant *in vitro* organogenesis. Sci. Rep. 6, 30746. 10.1038/srep30746 27478033 PMC4967872

[B13] Cleary M. A. Narcisi R. Focke K. van der Linden R. Brama P. A. van Osch G. J. (2016). Expression of CD105 on expanded mesenchymal stem cells does not predict their chondrogenic potential. Osteoarthr. Cartil. 24, 868–872. 10.1016/j.joca.2015.11.018 26687821

[B14] Crisan M. Yap S. Casteilla L. Chen C. W. Corselli M. Park T. S. (2008). A perivascular origin for mesenchymal stem cells in multiple human organs. Cell Stem Cell 3, 301–313. 10.1016/j.stem.2008.07.003 18786417

[B15] De Pieri A. Rana S. Korntner S. Zeugolis D. I. (2020). Seaweed polysaccharides as macromolecular crowding agents. Int. J. Biol. Macromol. 164, 434–446. 10.1016/j.ijbiomac.2020.07.087 32679331

[B16] de Soure A. M. Fernandes-Platzgummer A. da Silva C. L. Cabral J. M. (2016). Scalable microcarrier-based manufacturing of mesenchymal stem/stromal cells. J. Biotechnol. 236, 88–109. 10.1016/j.jbiotec.2016.08.007 27527397

[B17] Deans R. J. Moseley A. B. (2000). Mesenchymal stem cells: Biology and potential clinical uses. Exp. Hematol. 28, 875–884. 10.1016/s0301-472x(00)00482-3 10989188

[B18] Deschaseaux F. Gindraux F. Saadi R. Obert L. Chalmers D. Herve P. (2003). Direct selection of human bone marrow mesenchymal stem cells using an anti-CD49a antibody reveals their CD45med,low phenotype. Br. J. Haematol. 122, 506–517. 10.1046/j.1365-2141.2003.04469.x 12877680

[B19] Di Nicola M. Carlo-Stella C. Magni M. Milanesi M. Longoni P. D. Matteucci P. (2002). Human bone marrow stromal cells suppress T-lymphocyte proliferation induced by cellular or nonspecific mitogenic stimuli. Blood 99, 3838–3843. 10.1182/blood.v99.10.3838 11986244

[B20] Dominici M. Le Blanc K. Mueller I. Slaper-Cortenbach I. Marini F. Krause D. (2006). Minimal criteria for defining multipotent mesenchymal stromal cells. The International Society for Cellular Therapy position statement. Cytotherapy 8, 315–317. 10.1080/14653240600855905 16923606

[B21] Elgaz S. Kuçi Z. Kuçi S. Bönig H. Bader P. (2019). Clinical use of mesenchymal stromal cells in the treatment of acute graft-versus-host disease. Transfus. Med. Hemother 46, 27–34. 10.1159/000496809 31244579 PMC6558336

[B22] Gaspar D. Fuller K. P. Zeugolis D. I. (2019). Polydispersity and negative charge are key modulators of extracellular matrix deposition under macromolecular crowding conditions. Acta Biomater. 88, 197–210. 10.1016/j.actbio.2019.02.050 30831324

[B23] Gattazzo F. Urciuolo A. Bonaldo P. (2014). Extracellular matrix: A dynamic microenvironment for stem cell niche. Biochim. Biophys. Acta 1840, 2506–2519. 10.1016/j.bbagen.2014.01.010 24418517 PMC4081568

[B24] Graceffa V. Zeugolis D. I. (2019). Carrageenan enhances chondrogenesis and osteogenesis in human bone marrow stem cell culture. Eur. Cell Mater 37, 310–332. 10.22203/eCM.v037a19 31038192

[B25] Hagmann S. Frank S. Gotterbarm T. Dreher T. Eckstein V. Moradi B. (2014). Fluorescence activated enrichment of CD146+ cells during expansion of human bone-marrow derived mesenchymal stromal cells augments proliferation and GAG/DNA content in chondrogenic media. BMC Musculoskelet. Disord. 15, 322. 10.1186/1471-2474-15-322 25262357 PMC4196082

[B26] Hawkes P. W. (2015). Fetal bovine serum: Geographic origin and regulatory relevance of viral contamination. Bioresour. Bioprocess 2, 34. 10.1186/s40643-015-0063-7

[B27] Heiskanen A. Satomaa T. Tiitinen S. Laitinen A. Mannelin S. Impola U. (2007). N-glycolylneuraminic acid xenoantigen contamination of human embryonic and mesenchymal stem cells is substantially reversible. Stem Cells 25, 197–202. 10.1634/stemcells.2006-0444 17008421

[B28] Honn K. V. Singley J. A. Chavin W. (1975). Fetal bovine serum: A multivariate standard. Proc. Soc. Exp. Biol. Med. 149, 344–347. 10.3181/00379727-149-38804 1153408

[B29] Horwitz E. M. Gordon P. L. Koo W. K. Marx J. C. Neel M. D. McNall R. Y. (2002). Isolated allogeneic bone marrow-derived mesenchymal cells engraft and stimulate growth in children with osteogenesis imperfecta: Implications for cell therapy of bone. Proc. Natl. Acad. Sci. U. S. A. 99, 8932–8937. 10.1073/pnas.132252399 12084934 PMC124401

[B30] Jaatinen T. Laine J. (2007). Isolation of mononuclear cells from human cord blood by Ficoll-Paque density gradient. Curr. Protoc. Stem Cell Biol. Chapter 2, Unit 2A.1. 10.1002/9780470151808.sc02a01s1 18785173

[B31] Jin L. T. Hwang S. Y. Yoo G. S. Choi J. K. (2004). Sensitive silver staining of protein in sodium dodecyl sulfate-polyacrylamide gels using an azo dye, calconcarboxylic acid, as a silver-ion sensitizer. Electrophoresis 25, 2494–2500. 10.1002/elps.200306002 15300767

[B32] Jorgensen C. Djouad F. Apparailly F. Noël D. (2003). Engineering mesenchymal stem cells for immunotherapy. Gene Ther. 10, 928–931. 10.1038/sj.gt.3302019 12732877

[B33] Jung S. Panchalingam K. M. Rosenberg L. Behie L. A. (2012). *Ex vivo* expansion of human mesenchymal stem cells in defined serum-free media. Stem Cells Int. 2012, 1–21. 10.1155/2012/123030 PMC3356989 22645619

[B34] Kakabadze Z. Chakhunashvili D. Gogilashvili K. Ediberidze K. Chakhunashvili K. Kalandarishvili K. (2019). Bone marrow stem cell and decellularized human amniotic membrane for the treatment of nonhealing wound after radiation therapy. Exp. Clin. Transpl. 17, 92–98. 10.6002/ect.MESOT2018.O29 30777530

[B35] Karnieli O. Friedner O. M. Allickson J. G. Zhang N. Jung S. Fiorentini D. (2017). A consensus introduction to serum replacements and serum-free media for cellular therapies. Cytotherapy 19, 155–169. 10.1016/j.jcyt.2016.11.011 28017599

[B36] Kawasaki-Oyama R. Braile D. Caldas H. Leal J. Goloni-Bertollo E. Pavarino-Bertelli E. (2008). Blood mesenchymal stem cell culture from the umbilical cord with and without Ficoll-Paque density gradient method. Rev. Bras. Cir. Cardiovasc 23, 29–34. 10.1590/s0102-76382008000100006 18719825

[B37] Kinzebach S. Bieback K. (2013). Expansion of mesenchymal stem/stromal cells under xenogenic-free culture conditions. Adv. Biochem. Eng. Biotechnol. 129, 33–57. 10.1007/10_2012_134 22777242

[B38] Kumar P. Satyam A. Cigognini D. Pandit A. Zeugolis D. I. (2018). Low oxygen tension and macromolecular crowding accelerate extracellular matrix deposition in human corneal fibroblast culture. J. Tissue Eng. Regen. Med. 12, 6–18. 10.1002/term.2283 27592127

[B39] Kumar P. Satyam A. Fan X. Collin E. Rochev Y. Rodriguez B. J. (2015a). Macromolecularly crowded *in vitro* microenvironments accelerate the production of extracellular matrix-rich supramolecular assemblies. Sci. Rep. 5 (8729), 8729. 10.1038/srep08729 25736020 PMC4348624

[B40] Kumar P. Satyam A. Fan X. Rochev Y. Rodriguez B. J. Gorelov A. (2015b). Accelerated development of supramolecular corneal stromal-like assemblies from corneal fibroblasts in the presence of macromolecular crowders. Tissue Eng. Part C Methods 21, 660–670. 10.1089/ten.TEC.2014.0387 25535812

[B41] L'Heureux N. Dusserre N. Konig G. Victor B. Keire P. Wight T. N. (2006). Human tissue-engineered blood vessels for adult arterial revascularization. Nat. Med. 12, 361–365. 10.1038/nm1364 16491087 PMC1513140

[B42] Lareu R. R. Subramhanya K. H. Peng Y. Benny P. Chen C. Wang Z. (2007). Collagen matrix deposition is dramatically enhanced *in vitro* when crowded with charged macromolecules: The biological relevance of the excluded volume effect. FEBS Lett. 581, 2709–2714. 10.1016/j.febslet.2007.05.020 17531987

[B43] Lee J. Y. Kang M. H. Jang J. E. Lee J. E. Yang Y. Choi J. Y. (2022). Comparative analysis of mesenchymal stem cells cultivated in serum free media. Sci. Rep. 12, 8620. 10.1038/s41598-022-12467-z 35597800 PMC9124186

[B44] Lee M. H. Goralczyk A. G. Kriszt R. Ang X. M. Badowski C. Li Y. (2016). ECM microenvironment unlocks Brown adipogenic potential of adult human bone marrow-derived MSCs. Sci. Rep. 6, 21173. 10.1038/srep21173 26883894 PMC4756694

[B45] Levengood S. L. Zhang M. (2014). Chitosan-based scaffolds for bone tissue engineering. J. Mater Chem. B 2, 3161–3184. 10.1039/C4TB00027G 24999429 PMC4078888

[B46] Li X. Guo W. Zha K. Jing X. Wang M. Zhang Y. (2019). Enrichment of CD146(+) adipose-derived stem cells in combination with articular cartilage extracellular matrix scaffold promotes cartilage regeneration. Theranostics 9, 5105–5121. 10.7150/thno.33904 31410204 PMC6691381

[B47] Lv F. J. Tuan R. S. Cheung K. M. Leung V. Y. (2014). Concise review: The surface markers and identity of human mesenchymal stem cells. Stem Cells 32, 1408–1419. 10.1002/stem.1681 24578244

[B48] Mafi P. Hindocha S. Mafi R. Griffin M. Khan W. S. (2011). Adult mesenchymal stem cells and cell surface characterization - a systematic review of the literature. Open J. Orthop. 5, 253–260. 10.2174/1874325001105010253 PMC3178966 21966340

[B49] Mittal S. Chowhan R. K. Singh L. R. (2015). Macromolecular crowding: Macromolecules friend or foe. Biochim. Biophys. Acta 1850, 1822–1831. 10.1016/j.bbagen.2015.05.002 25960386

[B50] Najar M. Rodrigues R. M. Buyl K. Branson S. Vanhaecke T. Lagneaux L. (2014). Proliferative and phenotypical characteristics of human adipose tissue-derived stem cells: Comparison of Ficoll gradient centrifugation and red blood cell lysis buffer treatment purification methods. Cytotherapy 16, 1220–1228. 10.1016/j.jcyt.2014.05.021 25065636

[B51] O'Brien J. Wilson I. Orton T. Pognan F. (2000). Investigation of the Alamar Blue (resazurin) fluorescent dye for theassessment of mammalian cell cytotoxicity. Eur. J. Biochem. 267, 5421–5426. 10.1046/j.1432-1327.2000.01606.x 10951200

[B52] Okolicsanyi R. K. Camilleri E. T. Oikari L. E. Yu C. Cool S. M. van Wijnen A. J. (2015). Human mesenchymal stem cells retain multilineage differentiation capacity including neural marker expression after extended *in vitro* expansion. PLoS One 10, e0137255. 10.1371/journal.pone.0137255 26356539 PMC4565666

[B53] Patrikoski M. Lee M. H. C. Makinen L. Ang X. M. Mannerstrom B. Raghunath M. (2017). Effects of macromolecular crowding on human adipose stem cell culture in fetal bovine serum, human serum, and defined xeno-free/serum-free conditions. Stem Cells Int. 2017, 1–14. 10.1155/2017/6909163 PMC5390653 28465691

[B54] Pei M. He F. Kish V. L. (2011). Expansion on extracellular matrix deposited by human bone marrow stromal cells facilitates stem cell proliferation and tissue-specific lineage potential. Tissue Eng. Part A 17, 3067–3076. 10.1089/ten.TEA.2011.0158 21740327 PMC3226057

[B55] Prewitz M. C. Stißel A. Friedrichs J. Träber N. Vogler S. Bornhäuser M. (2015). Extracellular matrix deposition of bone marrow stroma enhanced by macromolecular crowding. Biomaterials 73, 60–69. 10.1016/j.biomaterials.2015.09.014 26398310

[B56] Raghunath M. Zeugolis D. (2021). Transforming eukaryotic cell culture with macromolecular crowding. Trends Biochem. Sci. 46, 805–811. 10.1016/j.tibs.2021.04.006 33994289

[B57] Rashid R. Lim N. S. Chee S. M. Png S. N. Wohland T. Raghunath M. (2014). Novel use for polyvinylpyrrolidone as a macromolecular crowder for enhanced extracellular matrix deposition and cell proliferation. Tissue Eng. Part C Methods 20, 994–1002. 10.1089/ten.TEC.2013.0733 24665935 PMC4241873

[B58] Roskelley C. D. Bissell M. J. (1995). Dynamic reciprocity revisited: A continuous, bidirectional flow of information between cells and the extracellular matrix regulates mammary epithelial cell function. Biochem. Cell Biol. 73, 391–397. 10.1139/o95-046 8703411

[B59] Russell K. C. Phinney D. G. Lacey M. R. Barrilleaux B. L. Meyertholen K. E. O'Connor K. C. (2010). *In vitro* high-capacity assay to quantify the clonal heterogeneity in trilineage potential of mesenchymal stem cells reveals a complex hierarchy of lineage commitment. Stem Cells 28, 788–798. 10.1002/stem.312 20127798

[B60] Sacchetti B. Funari A. Michienzi S. Di Cesare S. Piersanti S. Saggio I. (2007). Self-renewing osteoprogenitors in bone marrow sinusoids can organize a hematopoietic microenvironment. Cell 131, 324–336. 10.1016/j.cell.2007.08.025 17956733

[B61] Satyam A. Kumar P. Cigognini D. Pandit A. Zeugolis D. I. (2016). Low, but not too low, oxygen tension and macromolecular crowding accelerate extracellular matrix deposition in human dermal fibroblast culture. Acta Biomater. 44, 221–231. 10.1016/j.actbio.2016.08.008 27506127

[B62] Satyam A. Kumar P. Fan X. Gorelov A. Rochev Y. Joshi L. (2014). Macromolecular crowding meets tissue engineering by self-assembly: A paradigm shift in regenerative medicine. Adv. Mater 26, 3024–3034. 10.1002/adma.201304428 24505025

[B63] Selvaggi T. A. Walker R. E. Fleisher T. A. (1997). Development of antibodies to fetal calf serum with arthus-like reactions in human immunodeficiency virus-infected patients given syngeneic lymphocyte infusions. Blood 89, 776–779. 10.1182/blood.v89.3.776 9028307

[B64] Shendi D. Marzi J. Linthicum W. Rickards A. J. Dolivo D. M. Keller S. (2019). Hyaluronic acid as a macromolecular crowding agent for production of cell-derived matrices. Acta Biomater. 100, 292–305. 10.1016/j.actbio.2019.09.042 31568877

[B65] Siddappa R. Licht R. van Blitterswijk C. de Boer J. (2007). Donor variation and loss of multipotency during *in vitro* expansion of human mesenchymal stem cells for bone tissue engineering. J. Orthop. Res. 25, 1029–1041. 10.1002/jor.20402 17469183

[B66] Siegel G. Kluba T. Hermanutz-Klein U. Bieback K. Northoff H. Schäfer R. (2013). Phenotype, donor age and gender affect function of human bone marrow-derived mesenchymal stromal cells. BMC Med. 11, 146. 10.1186/1741-7015-11-146 23758701 PMC3694028

[B67] Sundin M. Ringdén O. Sundberg B. Nava S. Götherström C. Le Blanc K. (2007). No alloantibodies against mesenchymal stromal cells, but presence of anti-fetal calf serum antibodies, after transplantation in allogeneic hematopoietic stem cell recipients. Haematologica 92, 1208–1215. 10.3324/haematol.11446 17666368

[B68] Tekkatte C. Gunasingh G. P. Cherian K. M. Sankaranarayanan K. (2011). Humanized" stem cell culture techniques: The animal serum controversy. Stem Cells Int. 2011, 1–14. 10.4061/2011/504723 PMC3096451 21603148

[B69] Thorne J. T. Segal T. R. Chang S. Jorge S. Segars J. H. Leppert P. C. (2015). Dynamic reciprocity between cells and their microenvironment in reproduction. Biol. Reprod. 92, 25. 10.1095/biolreprod.114.121368 25411389 PMC4434933

[B70] Tsiapalis D. Kearns S. Kelly J. Zeugolis D. (2021). Growth factor and macromolecular crowding supplementation in human tenocyte culture. Biomaterials Biosyst. 1, 100009. 10.1016/j.bbiosy.2021.100009 PMC9934496 36825160

[B71] Tsiapalis D. Zeugolis D. (2021). It is time to crowd your cell culture media – physicochemical considerations with biological consequences. Biomaterials 275, 120943. 10.1016/j.biomaterials.2021.120943 34139505

[B72] van der Valk J. (2022). Fetal bovine serum - a cell culture dilemma. Science 375, 143–144. 10.1126/science.abm1317 35025663

[B73] van Helvert S. Storm C. Friedl P. (2018). Mechanoreciprocity in cell migration. Nat. Cell Biol. 20, 8–20. 10.1038/s41556-017-0012-0 29269951 PMC5943039

[B74] Varma M. J. Breuls R. G. Schouten T. E. Jurgens W. J. Bontkes H. J. Schuurhuis G. J. (2007). Phenotypical and functional characterization of freshly isolated adipose tissue-derived stem cells. Stem Cells Dev. 16, 91–104. 10.1089/scd.2006.0026 17348807

[B75] Wan Safwani W. K. Makpol S. Sathapan S. Chua K. H. (2011). The changes of stemness biomarkers expression in human adipose-derived stem cells during long-term manipulation. Biotechnol. Appl. Biochem. 58, 261–270. 10.1002/bab.38 21838801

[B76] Whitfield M. J. Lee W. C. Van Vliet K. J. (2013). Onset of heterogeneity in culture-expanded bone marrow stromal cells. Stem Cell Res. 11, 1365–1377. 10.1016/j.scr.2013.09.004 24103495

[B77] Wu C. C. Liu F. L. Sytwu H. K. Tsai C. Y. Chang D. M. (2016). CD146+ mesenchymal stem cells display greater therapeutic potential than CD146-cells for treating collagen-induced arthritis in mice. Stem Cell Res. Ther. 7, 23. 10.1186/s13287-016-0285-4 26841872 PMC4741021

[B78] Wu L. Cai X. Dong H. Jing W. Huang Y. Yang X. (2010). Serum regulates adipogenesis of mesenchymal stem cells via MEK/ERK-dependent PPARγ expression and phosphorylation. J. Cell Mol. Med. 14, 922–932. 10.1111/j.1582-4934.2009.00709.x 19243475 PMC3823124

[B79] Yang Y. Lin H. Shen H. Wang B. Lei G. Tuan R. S. (2018a). Mesenchymal stem cell-derived extracellular matrix enhances chondrogenic phenotype of and cartilage formation by encapsulated chondrocytes *in vitro* and *in vivo* . Acta Biomater. 69, 71–82. 10.1016/j.actbio.2017.12.043 29317369 PMC5831499

[B80] Yang Y. Ogando C. R. Wang See C. Chang T. Y. Barabino G. A. (2018b). Changes in phenotype and differentiation potential of human mesenchymal stem cells aging *in vitro* . Stem Cell Res. Ther. 9, 131. 10.1186/s13287-018-0876-3 29751774 PMC5948736

[B81] Yao L. Bestwick C. S. Bestwick L. A. Maffulli N. Aspden R. M. (2006). Phenotypic drift in human tenocyte culture. Tissue Eng. 12, 1843–1849. 10.1089/ten.2006.12.1843 16889514

[B82] Yoshimura K. Shigeura T. Matsumoto D. Sato T. Takaki Y. Aiba-Kojima E. (2006). Characterization of freshly isolated and cultured cells derived from the fatty and fluid portions of liposuction aspirates. J. Cell Physiol. 208, 64–76. 10.1002/jcp.20636 16557516

[B83] Yu G. Wu X. Dietrich M. A. Polk P. Scott L. K. Ptitsyn A. A. (2010). Yield and characterization of subcutaneous human adipose-derived stem cells by flow cytometric and adipogenic mRNA analyzes. Cytotherapy 12, 538–546. 10.3109/14653241003649528 20380539 PMC4346176

[B84] Yuan X. Liu Y. Bijonowski B. M. Tsai A.-C. Fu Q. Logan T. M. (2020). NAD+/NADH redox alterations reconfigure metabolism and rejuvenate senescent human mesenchymal stem cells *in vitro* . Commun. Biol. 3, 774. 10.1038/s42003-020-01514-y 33319867 PMC7738682

[B85] Zeiger A. S. Loe F. C. Li R. Raghunath M. Van Vliet K. J. (2012). Macromolecular crowding directs extracellular matrix organization and mesenchymal stem cell behavior. PLoS One 7, e37904. 10.1371/journal.pone.0037904 22649562 PMC3359376

[B86] Zeugolis D. (2021). Bioinspired *in vitro* microenvironments to control cell fate: Focus on macromolecular crowding. Am. J. Physiol. Cell Physiol. 320, C842–C849. 10.1152/ajpcell.00380.2020 33656930

[B87] Zhang J. Huang X. Wang H. Liu X. Zhang T. Wang Y. (2015). The challenges and promises of allogeneic mesenchymal stem cells for use as a cell-based therapy. Stem Cell Res. Ther. 6, 234. 10.1186/s13287-015-0240-9 26620426 PMC4665863

