# Supplementary material for: Macromolecular crowding in animal component-free, xeno-free and foetal bovine serum media for human bone marrow mesenchymal stromal cell expansion and differentiation
Source: Front Bioeng Biotechnol. 2023 Mar 6;11:1136827. doi: 10.3389/fbioe.2023.1136827 (PMC10025396; doi:10.3389/fbioe.2023.1136827)

# Supplemental Information

# Title

Macromolecular crowding in animal component-free, xeno-free and foetal bovine serum media for human bone marrow mesenchymal stromal cell expansion and differentiation

# Authors

Stefanie H. Korntner † (1), Alessia Di Nubila † (1), Diana Gaspar (1), Dimitrios I. Zeugolis * (1, 2)

# Affiliations

(1) Regenerative, Modular & Developmental Engineering Laboratory (REMODEL) and Science Foundation Ireland (SFI) Centre for Research in Medical Devices (CÚRAM), Biomedical Sciences Building, University of Galway, Galway, Ireland

(2) Regenerative, Modular & Developmental Engineering Laboratory (REMODEL), Charles Institute of Dermatology, Conway Institute of Biomolecular & Biomedical Research and School of Mechanical & Materials Engineering, University College Dublin (UCD), Dublin, Ireland

† These authors contributed equally to this work

* Corresponding Author: Dimitrios I. Zeugolis, REMODEL, UCD, Ireland

Telephone: +353 (0) 17 16 18 84; Email: dimitrios.zevgolis@ucd.ie

**Supplemental Table S1:** Different media conditions used for hBMSCs expansion up to p4.

| **Media** | **MMC** | **Acronym** |
| --- | --- | --- |
| MesenCult™-ACF Plus | - | ACF -MMC |
| MesenCult™-ACF Plus | + | ACF +MMC |
| MSC NutriStem® XF Medium | - | XF -MMC |
| MSC NutriStem® XF Medium | + | XF +MMC |
| α-MEM + 10 % FBS | - | FBS -MMC |
| α-MEM + 10 % FBS | + | FBS +MMC |

**Supplemental Table S2:** Primary antibodies for flow cytometry analysis and immunocytochemistry

| **Antibody** | **Supplier** | **Catalogue number** | **Concentration** |
| --- | --- | --- | --- |
| FITC Mouse Anti-Human CD90 | BD Biosciences | 51-9007657 | 1:20 |
| PE Mouse Anti-Human CD44 | BD Biosciences | 51-9007656 | 1:20 |
| APC Mouse Anti-Human CD73 | BD Biosciences | 51-9007649 | 1:20 |
| PerCP-Cy™5.5 Mouse Anti-Human CD105 | BD Biosciences | 51-9007648 | 1:20 |
| Anti-CD146 PE | Invitrogen | 12-1469-41 | 0.025 μg/μl |
| Anti—Hu CD31 (PECAM-1) | Invitrogen | 11-0319-41 | 0.1 μg/μl |
| Anti—Hu CD45 PerCP-EF710 | Invitrogen | 46-0459-41 | 0.025 μg/μl |
| hMSC Positive Cocktail Isotype Control: mIgG1, κ FITC (Clone: X40); mIgG1, κ PerCP-Cy5.5 (Clone: X40); mIgG1, κ APC (Clone: X40) | BD Biosciences | 51-9007664 | 1:5 |
| PE Mouse IgG2b, k Isotype Control (PE) | BD Biosciences | 51-9007655 | 1:20 |
| Mouse IgG1 kappa Isotype Control, FITC | Invitrogen | 11-4714-41 | 0.2 μg/μl |
| Mouse IgG1 kappa Isotype Control, PerCP-eFluor 710 | Invitrogen | 46-4714-80 | 0.2 μg/μl |
| Mouse IgG1 kappa Isotype Control, PE | Invitrogen | 12-4714-41 | 0.1 μg/ μl |
| Rabbit anti-collagen type I | Abcam | ab34719 | 2.5 μl/ml |
| AlexaFluor® 488  goat anti-rabbit | Molecular Probes®, Life Technologies | A11008 | 2.5 μl/ml |

**Supplemental Table S3:** Different MMC and media conditions used for adipogenic, osteogenic, and chondrogenic differentiation of hBMSCs, respectively, in p4.

| **Expansion media** | **MMC during expansion** | **MMC during differentiation** | **Acronym** |
| --- | --- | --- | --- |
| MesenCult™-ACF Plus | **-** | **-** | ACF **-/-** |
|  |  | **+** | ACF **-/+** |
| MesenCult™-ACF Plus | **+** | **-** | ACF **/-** |
|  |  | **+** | ACF **+/+** |
| MSC NutriStem® XF Medium | **-** | **-** | XF -/- |
|  |  | **+** | XF -/+ |
| MSC NutriStem® XF Medium | **+** | **-** | XF +/- |
|  |  | **+** | XF +/+ |
| α-MEM + 10 % FBS | **-** | **-** | FBS -/- |
|  |  | **+** | FBS -/+ |
| α-MEM + 10% FBS | **+** | **-** | FBS +/- |
|  |  | **+** | FBS +/+ |

**Supplemental Table S4:** Donor 1 hBMSCs were evaluated for positive (CD73, CD90, CD105, CD44, CD146) and negative (CD45, CD31) markers in p0 and p4, after 10 days of culture in ACF, FBS and XF media, without or with MMC.

| **Donor 1** | | **p0** | **p4** | | | | | |
| --- | --- | --- | --- | --- | --- | --- | --- | --- |
|  |  | **ACF -MMC** | **FBS -MMC** | **FBS +MMC** | **XF -MMC** | **XF +MMC** | **ACF -MMC** | **ACF +MMC** |
| **Positive markers**  **(% positive cells)** | **CD90** | 99.0 | 99.9 | 100.0 | 99.9 | 97.9 | 99.9 | 99.2 |
|  | **CD44** | 93.6 | 93.9 | 99.6 | 92.3 | 93.4 | 89.5 | 85.3 |
|  | **CD73** | 96.0 | 99.4 | 99.8 | 99.1 | 96.7 | 97.2 | 94.4 |
|  | **CD105** | 81.5 | 51.0 | 81.0 | 43.0 | 90.4 | 84.9 | 49.1 |
|  | **CD146** | 72.1 | 9.27 | 86.1 | 67.9 | 44.1 | 70.6 | 44.2 |
| **Negative markers**  **(% positive cells)** | **CD31** | 8.61 | 21.6 | 6.779 | 14.2 | 44.4 | 38.5 | 16.0 |
|  | **CD45** | 4.93 | 3.1 | 0.022 | 0.5 | 0.043 | 0.58 | 0.36 |

**Supplemental Table S5:** Donor 2 hBMSCs were evaluated for positive (CD73, CD90, CD105, CD44, CD146) and negative (CD45, CD31) markers in p0 and p4, after 10 days of culture in ACF, FBS and XF media, without or with MMC.

| **Donor 2** | | **p0** | **p4** | | | | | |
| --- | --- | --- | --- | --- | --- | --- | --- | --- |
|  |  | **ACF -MMC** | **FBS -MMC** | **FBS +MMC** | **XF -MMC** | **XF +MMC** | **ACF -MMC** | **ACF +MMC** |
| **Positive markers**  **(% positive cells)** | **CD90** | 99.6 | 98.6 | 96.7 | 93.2 | 99.4 | 98.5 | 94.9 |
|  | **CD44** | 92.5 | 99.2 | 81.4 | 92.2 | 95.5 | 99.6 | 92.2 |
|  | **CD73** | 97.3 | 88.0 | 87.3 | 90.6 | 90.3 | 95.9 | 87.7 |
|  | **CD105** | 86.3 | 53.4 | 64.5 | 87.9 | 87.3 | 36.6 | 19.7 |
|  | **CD146** | 54.7 | 11.4 | 1.18 | 39.7 | 42.7 | 60.5 | 32.7 |
| **Negative markers**  **(% positive cells)** | **CD31** | 0.81 | 33.8 | 14.9 | 87.0 | 83.1 | 1.58 | 28.7 |
|  | **CD45** | 29.5 | 44.2 | 53.6 | 89.5 | 76.7 | 1.35 | 45.0 |

**Supplemental Figure S1:** Flow cytometry of hBMSCs of donor 1 and 2 for positive (CD90, CD44, CD73, CD105, CD146) and negative mesenchymal stem cell surface markers (CD31 (endothelial) and CD45 (haematopoietic)) at p0 **(A-B)** and at p4 **(C-D)** expanded with or without MMC in FBS, XF and ACF media.


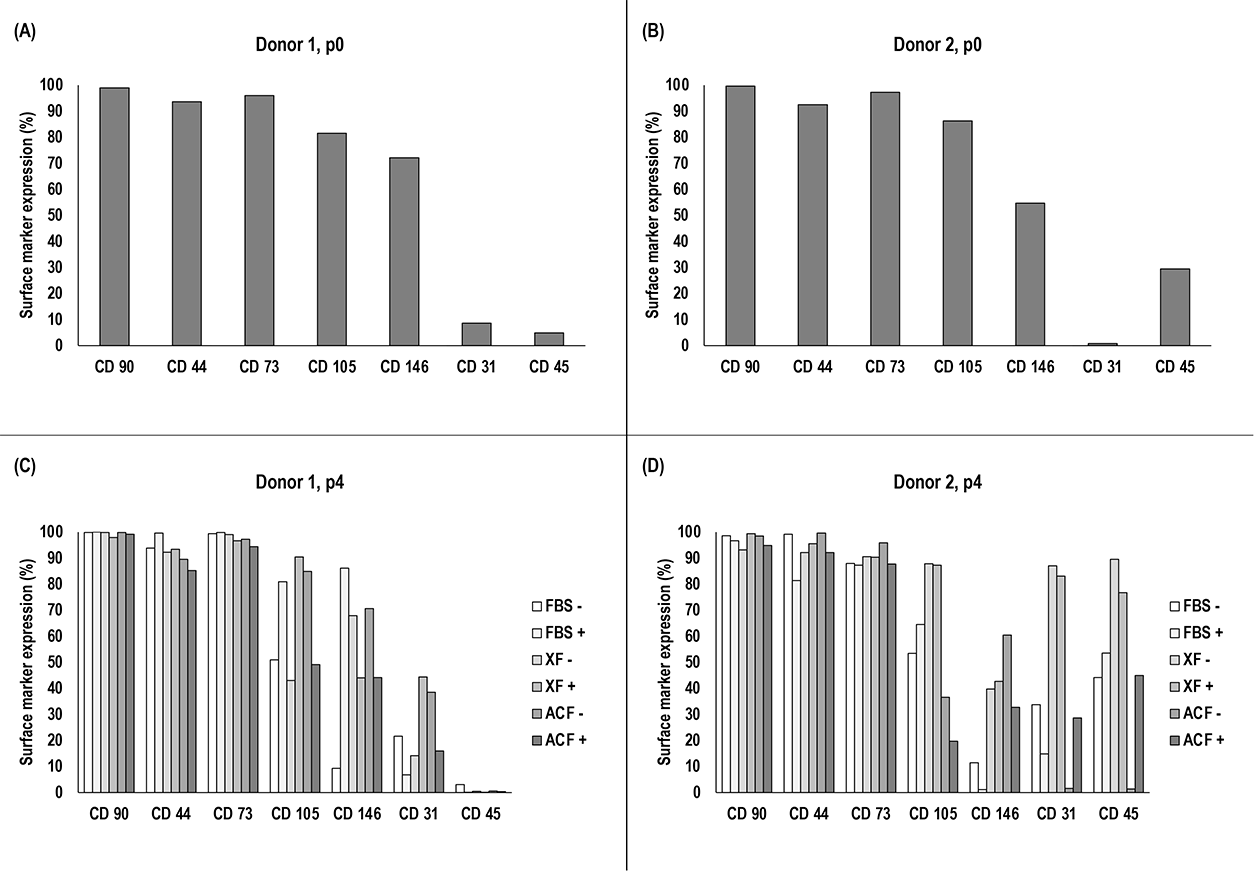


**Supplemental Figure S2:** Flow cytometry analysis of donor 1 **(A)** and donor 2 **(B)** hBMSCs at p0 after isolation from fresh human bone marrow with ACF (-MMC) media. Orange: isotype control; magenta: surface marker.


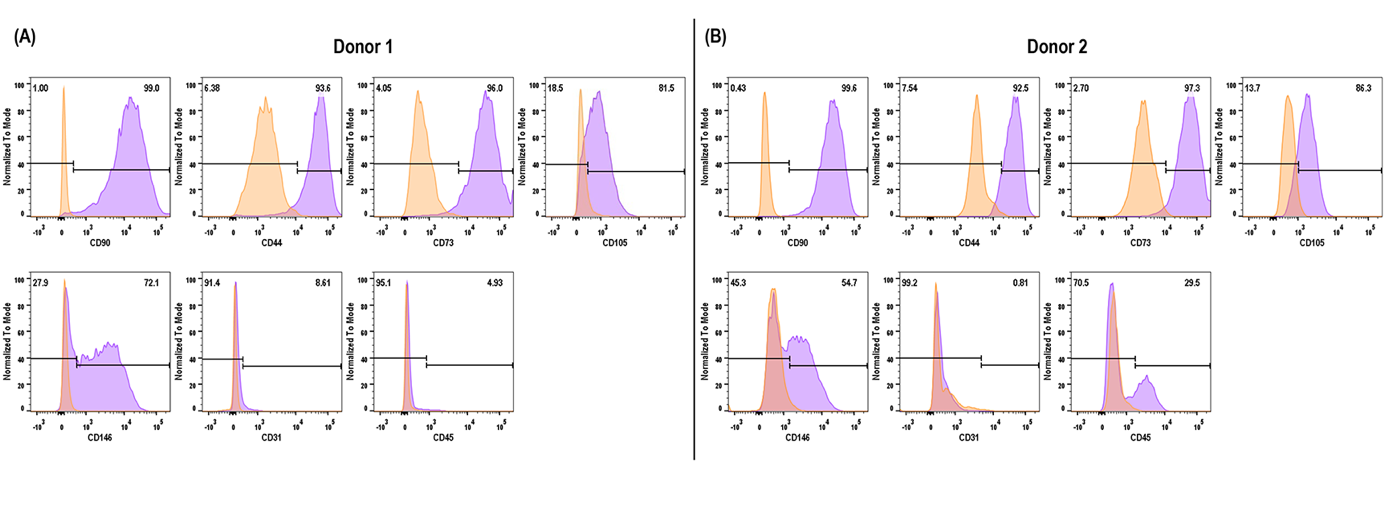


**Supplemental Figure S3:** Flow cytometry analysis of donor 1 hBMSCs expanded with or without MMC in FBS **(A-B)**, ACF **(C-D)**, and XF **(E-F)** media, at p4 after 10 days of culture. Orange: isotype control; magenta: surface marker.


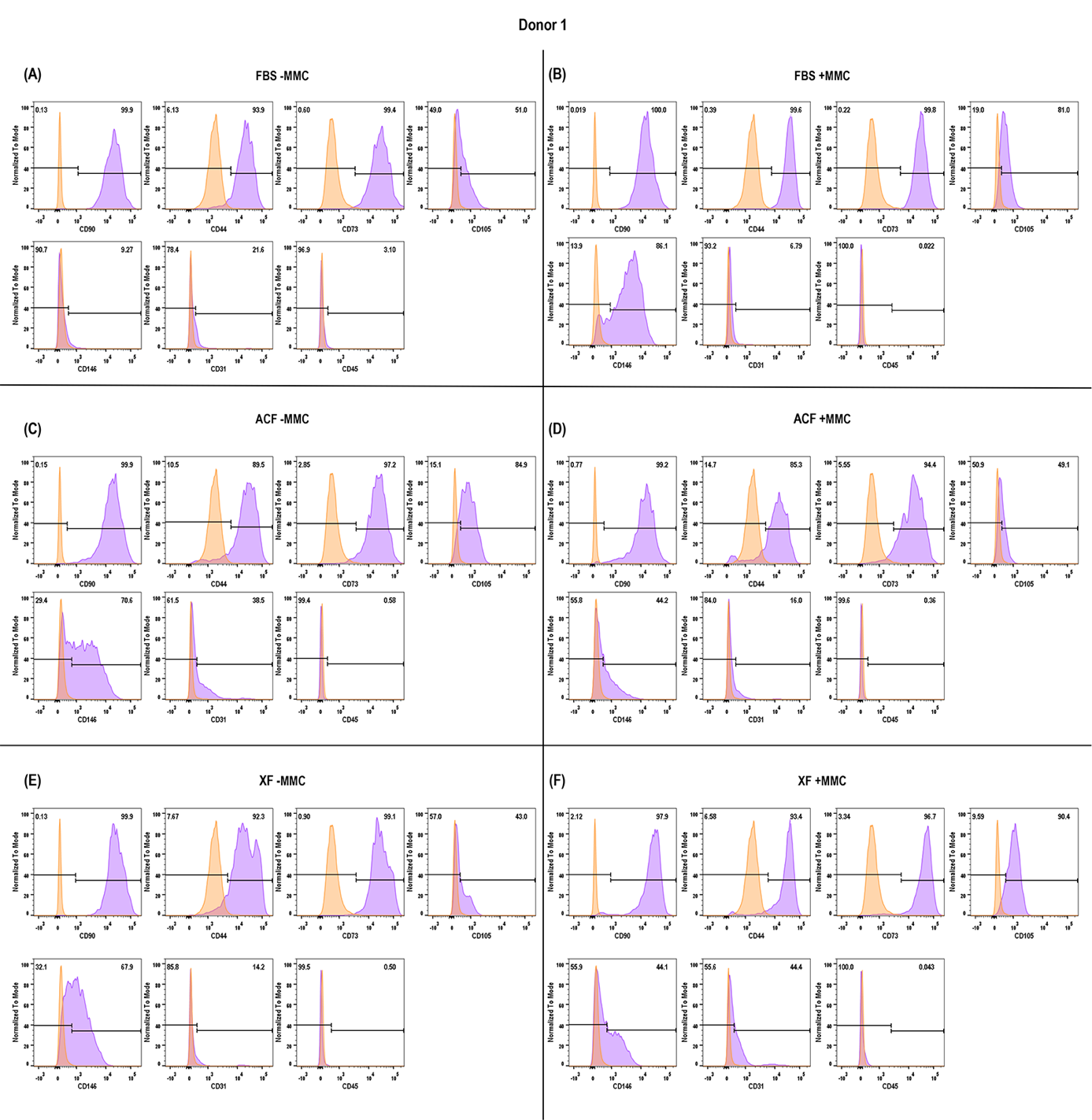


**Supplemental Figure S4:** Flow cytometry analysis of donor 2 hBMSCs expanded with or without MMC in FBS **(A-B)**, ACF **(C-D)**, and XF **(E-F)** media, at p4 after 10 days of culture. Orange: isotype control; magenta: surface marker.


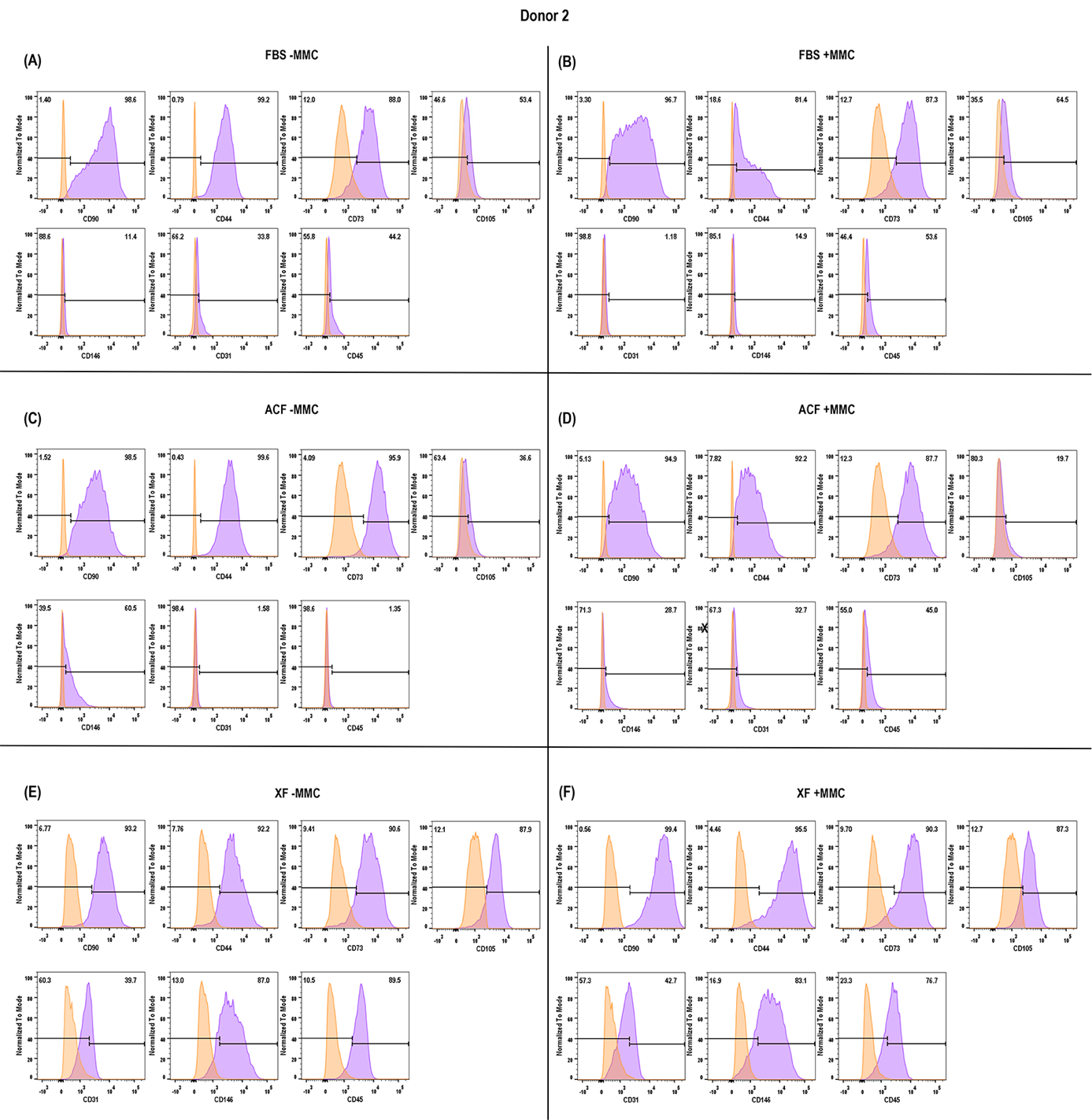


**Supplemental Figure S5:** Cell morphology of donor 1 **(A)** and donor 2 **(B)** hBMSCs expanded with or without MMC in FBS, XF and ACF media in p4 after 4 and 10 days of culture. Experiments were performed in 3 technical replicates. Scale bars: 100 μm.


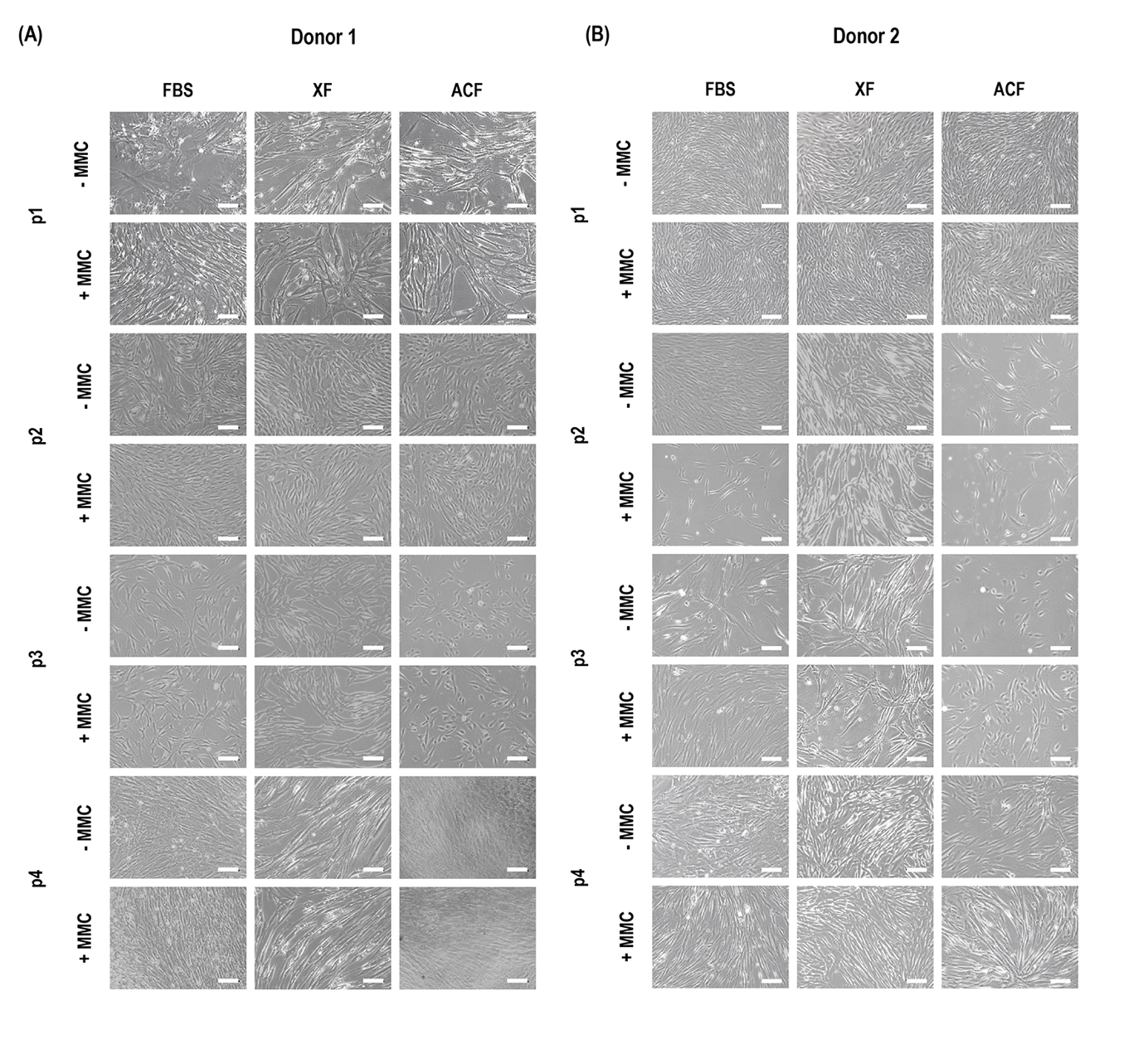


**Supplemental Figure S6:** Cell viability and corresponding semi-quantitative analysis of donor 1 **(A-B)** and donor 2 **(C-D)** hBMSCs expanded with or without MMC in FBS, XF, and ACF media in p4 after 4 and 10 days of culture. Experiments were performed in 3 technical replicates. Scale bars: 100 μm.


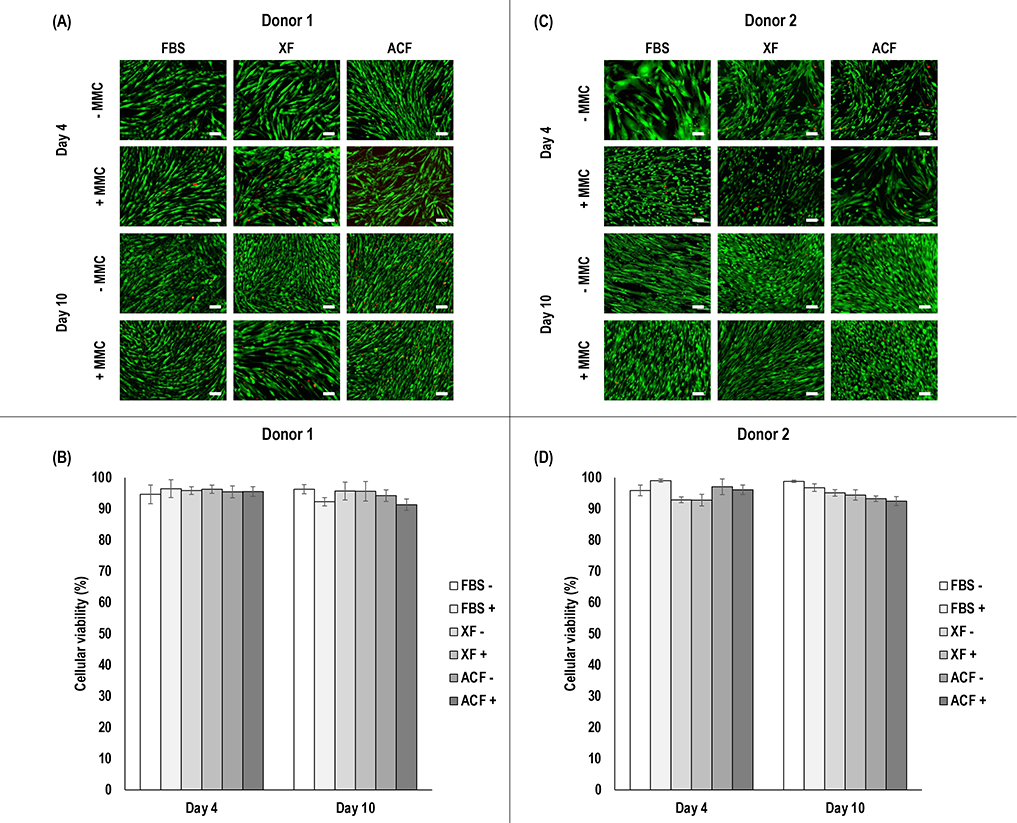


**Supplemental Figure S7:** Cell proliferation **(A-B)** and metabolic activity **(C-D)** of hBMSCs of donor 1 and 2 at p4 after 4 and 10 days of culture, expanded with or without MMC in FBS, XF, and ACF media. Metabolic activity is expressed as percentage of reduced alamarBlue® values normalised to DNA concentration (μg/ml). Experiments were performed in 3 technical replicates. “#” indicates the lowest statistically significant value (p<0.05) at a given time point, whereas “*” indicates the highest statistically significant value (p<0.05) at a given time point. A black line with “*” indicates the groups with the highest statistically significant value (p<0.05) at a given time point.


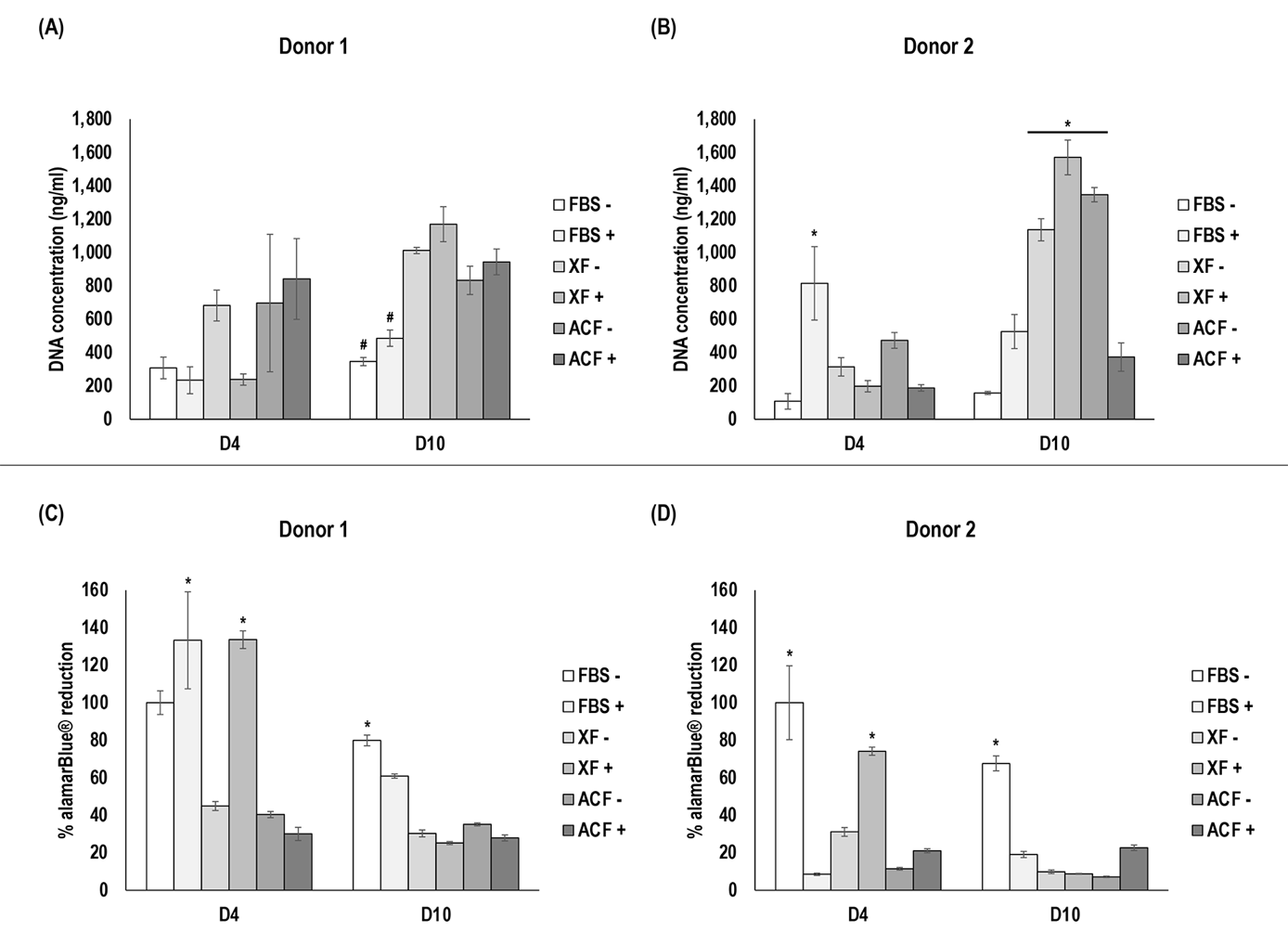


**Supplemental Figure S8:** Immunocytochemistry for collagen type I and complementary relative fluorescence intensity analysis of hBMSCs of donor 1 **(A-B)** and donor 2 **(C-D)** at p4 after 4 and 10 days of culture, expanded with or without MMC in FBS, XF, and ACF media. Analysis was performed with the ImageJ software and values were normalised to cell number. Experiments were performed in 3 technical replicates. “#” indicates the lowest significant value (p<0.05) at a given time point, whereas “*” indicates the highest significant value (p<0.05) at a given time point. Scale bar: 100 μm.


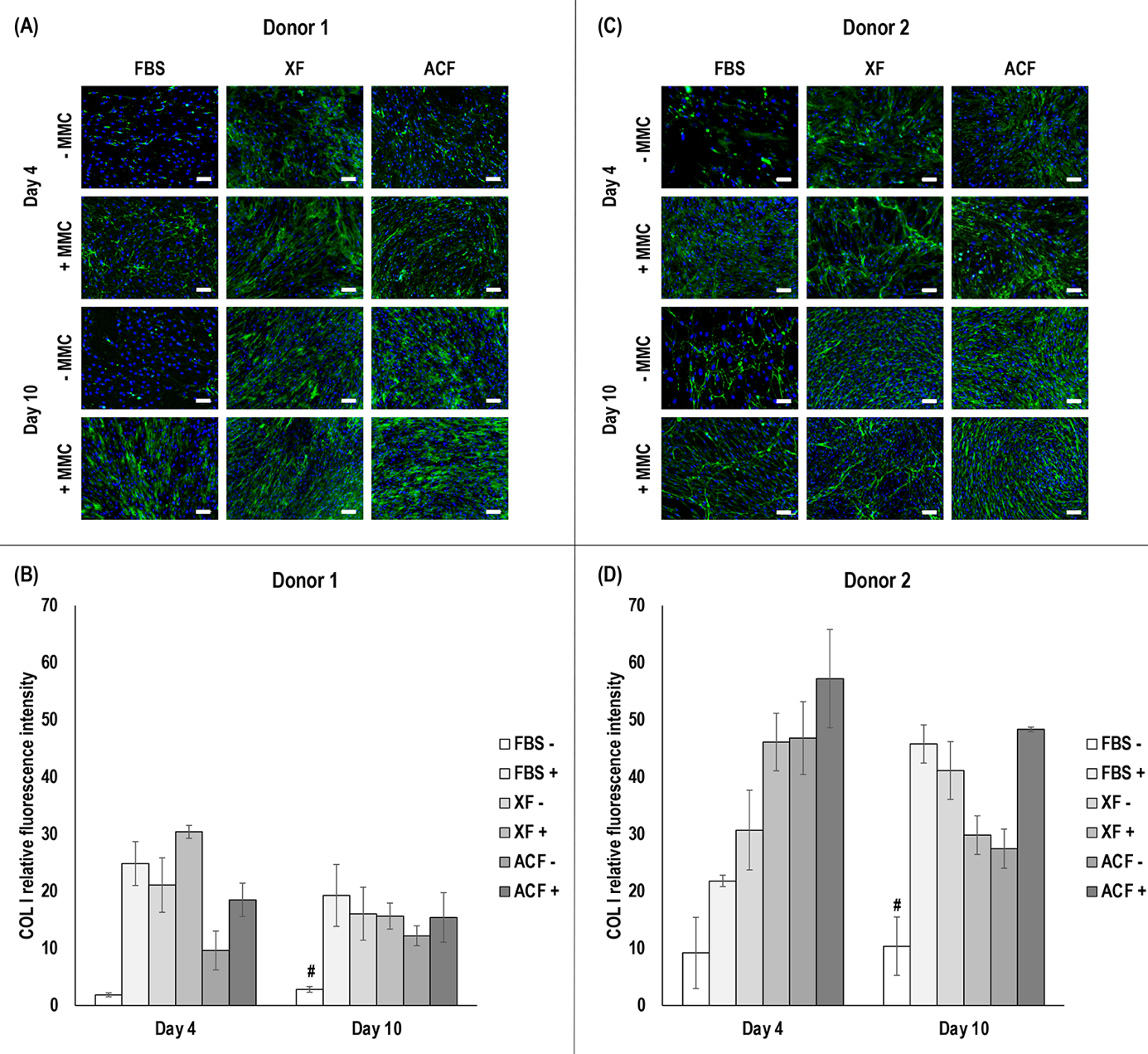


**Supplemental Figure S9:** Phase contrast images of hBMSCs of donor 1 **(A)** and donor 2 **(B)**, expanded with or without MMC in FBS, XF, and ACF media, and differentiated with adipogenic induction media with or without MMC supplementation at p4. Experiments were performed in 3 technical replicates. Scale bar: 100 μm.


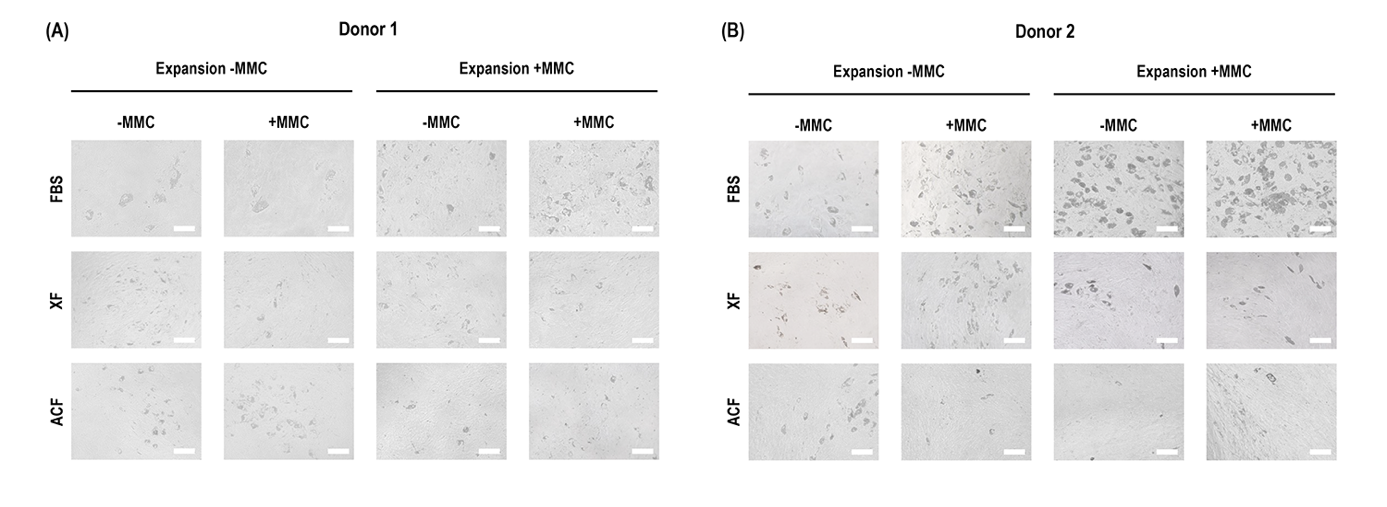


**Supplemental Figure S10:** Phase contrast images of hBMSCs of donor 1 **(A)** and donor 2 **(B)**, expanded with or without MMC in FBS, XF, and ACF media, and differentiated with osteogenic induction media with or without MMC supplementation at p4. Experiments were performed in 3 technical replicates. Scale bar: 100 μm.


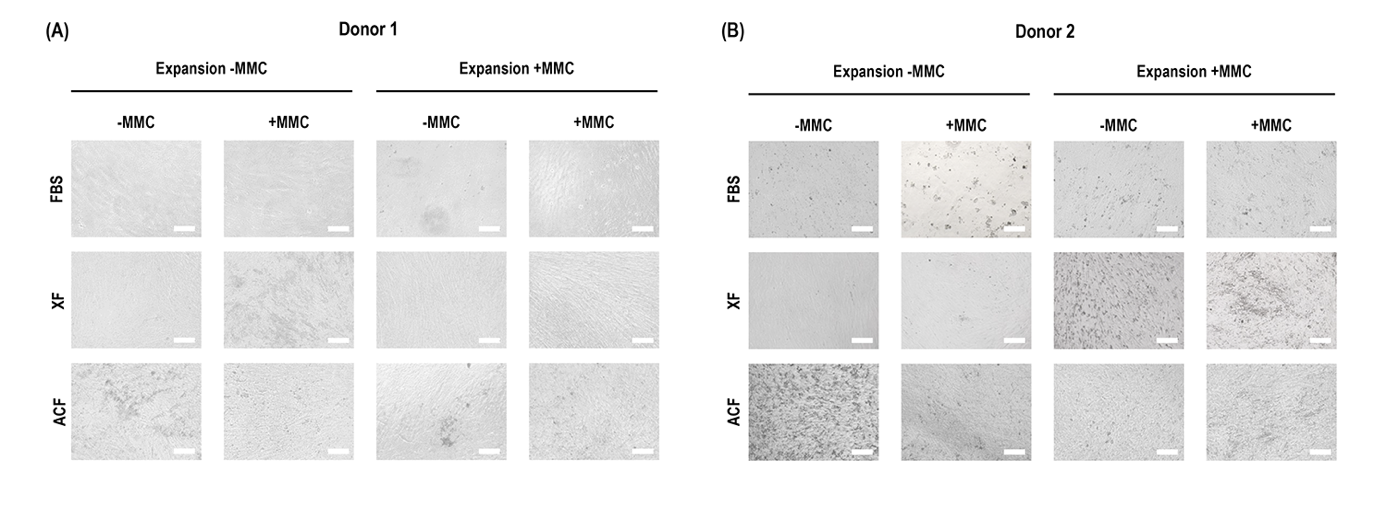


**Supplemental Figure S11:** SDS-PAGE **(A)** of XF and ACF attachment (coating) solutions only revealed no protein bands. Immunofluorescent staining **(B)** of XF and ACF coatings only showed no positive staining for collagen type I (COL1) and fibronectin (FN). Scale bar: 100 μm.


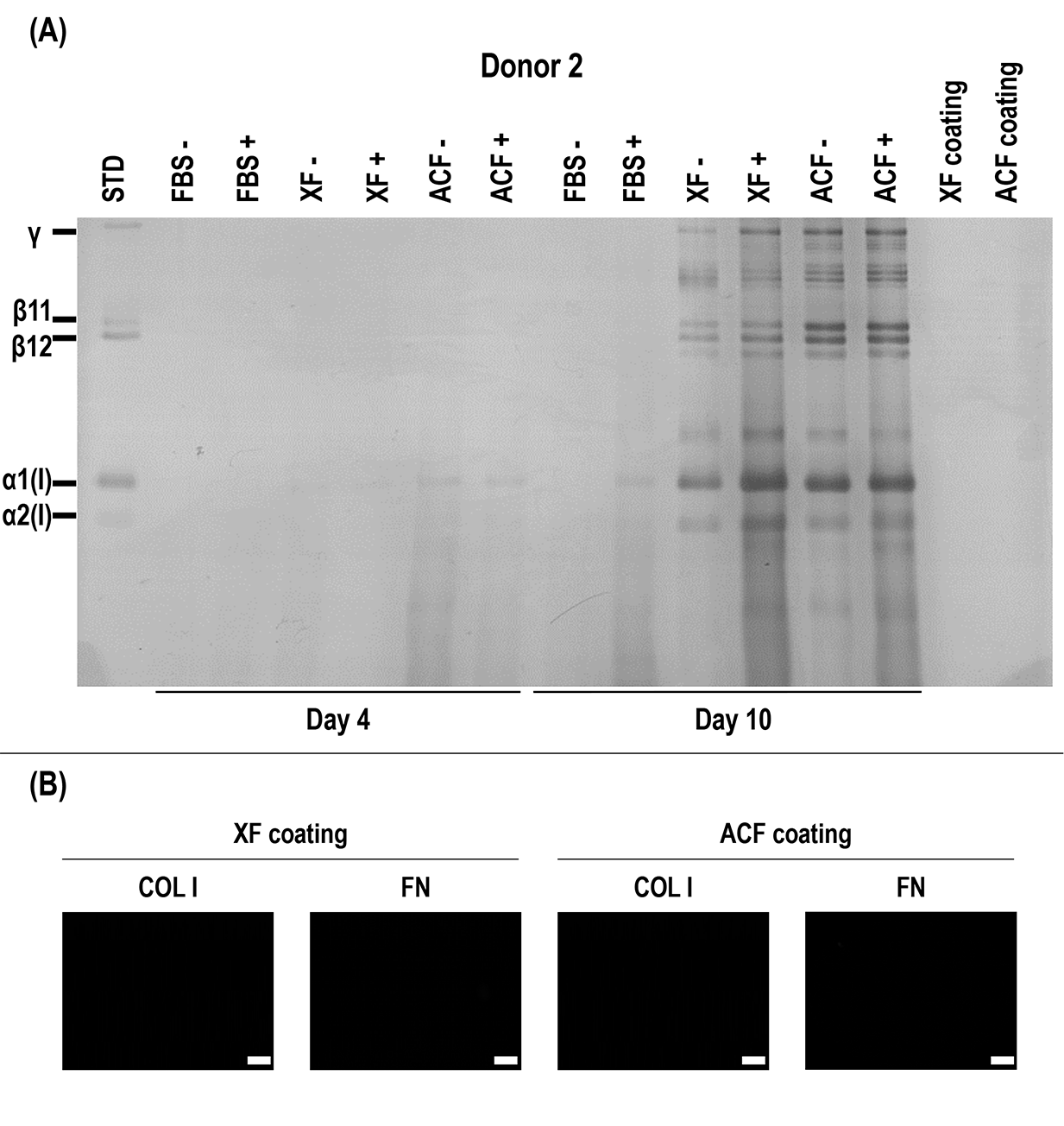

Supplement: Supplementary file 1 [file DataSheet1.docx]
